# Tricholides A and B and Unnarmicin D: New Hybrid PKS-NRPS Macrocycles Isolated from an Environmental Collection of *Trichodesmium thiebautii*

**DOI:** 10.3390/md15070206

**Published:** 2017-06-30

**Authors:** Matthew J. Bertin, Alexandre F. Roduit, Jiadong Sun, Gabriella E. Alves, Christopher W. Via, Miguel A. Gonzalez, Paul V. Zimba, Peter D. R. Moeller

**Affiliations:** 1 Department of Biomedical and Pharmaceutical Sciences, College of Pharmacy, University of Rhode Island, Kingston, RI 02881, USA; aroduit@my.uri.edu (A.F.R.); jiadong_sun@my.uri.edu (J.S.); gabriella_alves@my.uri.edu (G.E.A.); Christopher_via@my.uri.edu (C.W.V.); miggyg6@my.uri.edu (M.A.G.); 2 Center for Coastal Studies and Department of Life Sciences, Texas A&M Corpus Christi, 6300 Ocean Drive, Corpus Christi, TX 78412, USA; paul.zimba@tamucc.edu; 3 Emerging Toxins Program, National Ocean Service/NOAA, Hollings Marine Laboratory, 331 Fort Johnson Road, Charleston, SC 29412, USA; peter.moeller@noaa.gov

**Keywords:** *Trichodesmium thiebautii*, macrocycle, depsipeptide, cyanobacteria

## Abstract

Bioassay-guided isolation of the lipophilic extract of *Trichodesmium thiebautii* bloom material led to the purification and structure characterization of two new hybrid polyketide-non-ribosomal peptide (PKS-NRPS) macrocyclic compounds, tricholides A and B (**1** and **2**). A third macrocyclic compound, unnarmicin D (**3**), was identified as a new depsipeptide in the unnarmicin family, given its structural similarity to the existing compounds in this group. The planar structures of **1**–**3** were determined using 1D and 2D NMR spectra and complementary spectroscopic and spectrometric procedures. The absolute configurations of the amino acid components of **1**–**3** were determined via acid hydrolysis, derivitization with Marfey’s reagent and HPLC-UV comparison to authentic amino acid standards. The absolute configuration of the 3-hydroxydodecanoic acid moiety in **3** was determined using a modified Mosher’s esterification procedure on a linear derivative of tricharmicin (**4**) and additionally by a comparison of ^13^C NMR shifts of **3** to known depsipeptides with *β*-hydroxy acid subunits. Tricholide B (**2**) showed moderate cytotoxicity to Neuro-2A murine neuroblastoma cells (EC_50_: 14.5 ± 6.2 μM).

## 1. Introduction

Marine cyanobacteria have been fertile grounds for the isolation of a diverse array of structurally intriguing secondary metabolites with a broad-spectrum of biological activities [1,2,3,4]. A notable class of these compounds are the macrocyclic hybrid polyketide-non-ribosomal peptides (PKS-NRPS). The hybrid macrocycles isolated from cyanobacteria display unique and rare structural elements and functionalities, such as the dichlorinated beta-hydroxy acid and thiazole carboxylic acid units in the peptolide lyngbyabellin [5] and a rare *N*-methyl enamide of sanctolide A [6]. Macrocycles have been prolific structures in drug discovery, perhaps most notably as antibiotics [7]. However, this class displays diverse bioactivities including antitumor [8], antimalarial [9] and neuroactive properties, including sodium channel inhibition [10].

Many cyanobacterial macrocycles are peptides and depsipeptides [11] derived from NRPS or mixed NRPS-PKS genetic architecture and encode proteins with the ability to incorporate l- and d-*α*-amino acids as well as *β*-hydroxy fatty acids [12,13]. *β*-hydroxyacyl incorporation has been observed in several microbial depsipeptides including the beauverolides [14,15,16] (3 and 4 residue macrocycles); the unnarmicins [17], the solonamides [18], the arthroamides [19], the turnagainolides [20], and ngercheumicin C, D and E [21] (five residue macrocycles); and scytonemide B [22] and kailuin A-H (six residue macrocycles) [23,24]. These macrocycles have generally displayed antibiotic activity [17], quorum sensing interference [18] and weak cytotoxicity [24].

In this work, we detail the isolation and structure characterization of three new additions (**1**–**3**) to the PKS-NRPS macrocycles from *Trichodesmium thiebautii* bloom biomass. *Trichodesmium* spp. have been a relatively underexplored source of secondary metabolites. The cyclic peptide trichamide was isolated from a cultured specimen of *Trichodesmium erythraeum* IMS101 [25]. Trichophycin A and the trichotoxins, chlorinated polyketides have been isolated from environmental collections of *Trichodesmium thiebautii* [26,27]. Two of these metabolites in the current work, tricholides A and B (**1** and **2**), represent a new class of polyketide macrolactones, each incorporating a single proline residue and predicted 2-methylhexanoic acid residue. The third compound, unnarmicin D, departs from previously described unnarmicins by featuring a 3-hydroxydodecanoic acid residue.

## 2. Results

### 2.1. Isolation and Structure Determination of ***1**–**3***

Bioassy-guided isolation of the lipophilic extract of *Trichodesmium thiebautii* bloom material using human colon cancer HCT-116 cells identified a mixed fraction that showed potent cytotoxicity at a single dose of 40 μg/mL. Subsequent purification of the fraction using HPLC resulted in the isolation of 1. HRESIMS analysis of **1** identified a pseudomolecular ion [M + H]^+^ at *m*/*z* 408.3113 suggesting a molecular formula of C_24_H_41_NO_4_ and five degrees of unsaturation. Examination of the ^13^C NMR, HSQC and HMBC spectra identified two signals consistent with that of ester or amide functionalities, two alkene signals, two oxymethine carbons, three methine carbons, eleven methylene signals, and four methyl signals, one of which was consistent with that of an *O*-methyl (*δ*_C_ 56.0).

**Figure 1:**
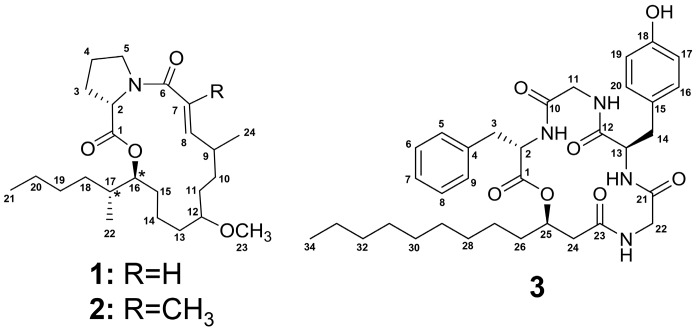
Structures of tricholide A (**1**), tricholide B (**2**) and unnarmicin D (**3**). The configuration of C-16 and C-17 in **1** and **2** is relative and noted by (*).

Examination of 1D and 2D NMR spectra (Figures S1–S7; Table 1) allowed for the construction of two partial structures. In the first partial structure, a methine proton signal (H-2, *δ*_H_ 4.50) showed COSY correlations to a diastereotopic methylene group (H-3a, *δ*_H_ 2.29 and H-3b, *δ*_H_ 2.10) and TOCSY correlations to two additional methylene groups (H_2_-4, *δ*_H_ 1.94; H-5a, *δ*_H_ 3.77; H-5b, *δ*_H_ 3.62). Examining the ^13^C NMR values of C-2, C-3, C-4, C-5 (*δ*_C_ 59.1, 31.8, 22.3, 46.7), we identified the first spin system as the amino acid proline. An HMBC correlation from H-2 to C-1 (*δ*_C_ 172.2) firmly established the *α* amino acid and satisfied two degrees of unsaturation. The second partial structure was comprised of a polarized olefin (C-7, *δ*_C_ 117.1; C-8, *δ*_C_ 151.1) that showed HMBC correlations to C-6 (*δ*_C_ 173.0) consistent with that of an *α*,*β*-unsaturated carbonyl functionality, satisfying the third and fourth degree of unsaturation. The distal proton in the olefin (H-8, *δ*_H_ 7.16) showed a COSY correlation to H-9 (*δ*_H_ 2.34). H-9 showed COSY correlations to a methyl signal (H-24, *δ*_H_ 1.04) and a methylene (H_2_-10, *δ*_H_ 1.36). The H_2_-10 methylene signal was correlated by TOCSY to second methylene group (H-11a, *δ*_H_ 1.62; H-11b, *δ*_H_ 1.43) and an oxymethine proton (H-12, *δ*_H_ 3.20). Bidirectional COSY correlations established three methylene groups between H-12 and a second deshielded oxymethine (H-16, *δ*_H_ 4.98). H-16 showed COSY correlations to H-17 (*δ*_H_ 1.60) and H-17 showed COSY correlations to a methyl (H_3_-22, *δ*_H_ 0.91) and a methylene group (H-18a, *δ*_H_ 1.34; H-18b, *δ*_H_ 1.10). Bidirectional COSY correlations from H_2_-18 to the terminal methyl H_3_-21 (*δ*_H_ 0.89) established two additional methylene groups in the alkyl chain (H-19a, *δ*_H_ 1.30; H-19b, *δ*_H_ 1.26; H_2_-20, *δ*_H_ 1.28) and completed the core of the second partial structure. An HMBC correlation from H_3_-23 (*δ*_H_ 3.27) to C-12 (*δ*_C_ 79.9) established an *O*-methyl group in the second partial structure. HMBC correlations from H_2_-5 to C-6 connected the two partial structures and the HMBC correlation from H-16 to C-1 demonstrated that **1** was a macrolactone featuring a proline residue and satisfied the final degree of unsaturation (Figure 1).

HRESIMS of **2** identified a pseudomolecular ion [M + H]^+^ at *m*/*z* 422.3270 suggesting a molecular formula of C_25_H_43_NO_4_ and five degrees of unsaturation as in **1**. The proton and carbon NMR spectra of **2** were nearly identical to **1** and the mass difference of 14 strongly suggested the addition of a CH_2_ group or methyl group instead of a proton in **2**. Examination of the ^1^H NMR, ^13^C NMR and 2D spectra of **2** (Figures S8–S14) showed a new singlet methyl signal (H_3_-25, *δ*_H_ 1.84) instead of the doublet signal H-7 (*δ*_H_ 7.16) in **1**. The ^13^C NMR spectrum showed one new signal (C-25, *δ*_C_ 14.8) (Table S1). HMBC correlations from H_3_-25 to C-6, C-7 and C-8 firmly established the methyl substitution at C-7 in **2**.

The relative configuration of the olefin in **1** was determined to be *E* by virtue of the large vicinal coupling constant between H-7 and H-8 (*J* = 15.5 Hz). The relative configuration between C-16 and C-17 was determined by examining the extracted ^1^H-^1^H coupling constant between H-16 and H-17. A large coupling constant of 10.6 Hz supported an *anti*-configuration. The relative configuration of **2** was identical to that of **1**.

The absolute configuration of the proline residues in **1** and **2** was determined by comparing the HPLC retention times of authentic l- and d/l-amino acid standards and the acid hydrolyzed constituents of **1** and **2** each reacted with Marfey’s reagent (L-FDVA). The hydrolyzate of both **1** and **2** showed retention times (min) that matched l-Pro (13.00; see Experimental Section and Figures S22 and S23). The small quantities of **1** and **2** isolated precluded further analysis of absolute configuration.

HRESIMS analysis of **3** identified a pseudomolecular ion [M + H]^+^ at *m*/*z* 623.3436 suggesting a molecular formula of C_34_H_46_N_4_O_7_ requiring 14 degrees of unsaturation. The peptidic nature of **3** was supported by five signals in the ^13^C NMR spectrum consistent with those of esters or amides (*δ*_C_ 168.2, 170.1, 170.7, 171.3, 172.4) (Figure S16) and the examination of the ^1^H NMR spectrum with doublets for four amide protons (*δ*_H_ 8.98, 8.61, 8.02 and 7.40) (Figure S15) accounting for five out of the seven oxygen atoms present and the four nitrogen atoms.

**Figure 2:**
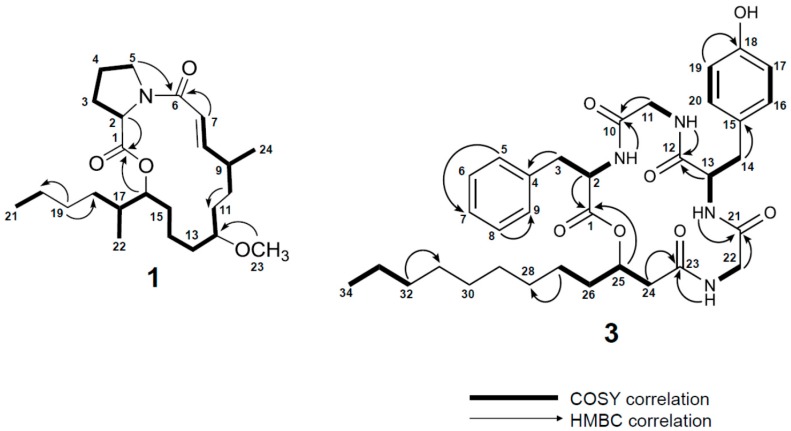
Selected 2D NMR correlations for **1** and **3**.

Analysis of 2D NMR data (Figures S17–S21) allowed two spin systems to be established (cf. Table 2 and Figure 2) as likely Gly residues. COSY data correlated an NH proton (NH-3, *δ*_H_ 8.98) to a methine proton (H-13, *δ*_H_ 4.13) which itself was correlated to a diastereotopic methylene group (H-14a, *δ*_H_ 3.02 and H-14b, *δ*_H_ 2.78). An HMBC correlation from the methylene group to a quaternary carbon (C-15, *δ*_C_ 127.8) and chemically equivalent carbons (C-16/20, *δ*_C_ 129.8) established a connection to an aromatic ring. The remaining carbons of the ring were comprised of chemically equivalent carbons (C-17/19, *δ*_C_ 115.1) bearing methine protons (H-17/19, *δ*_H_ 6.69, d, *J* = 8.5 Hz) and a quaternary carbon (C-20, *δ*_C_ 156.0) which strongly supported a Tyr residue. Additionally, COSY data correlated an NH proton (NH-1, *δ*_H_ 7.40) to a methine proton (H-2, *δ*_H_ 4.58) which itself was correlated to a second diastereotopic methylene group (H-3a, *δ*_H_ 3.15 and H-3b, *δ*_H_ 2.78). An HMBC correlation from this methylene group to a quaternary carbon (C-4, *δ*_C_ 137.6) and ^1^H-^1^H couplings between five methine protons on this second aromatic ring (C5–C9) supported a Phe residue. HMBC correlations from amino acid *α*-protons to their adjacent carbonyls allowed the assignment of four unmodified amino acids: Phe, Tyr, and two Gly residues (Figure 2). The presence of the two aromatic amino acids and five carbonyl functionalities accounted for 13 out of the 14 degrees of unsaturation. The final residue was comprised of a deshielded diastereotopic methylene group (H-24a, *δ*_H_ 2.60; H-24b, *δ*_H_ 2.19) which showed HMBC correlations to the C-23 carbonyl (*δ*_C_ 170.7) and an oxygen-bearing methine (C-25, *δ*_C_ 72.5). The oxymethine proton (H-25, *δ*_H_ 5.13) showed a COSY correlation to H_2_-26 (*δ*_H_ 1.54 and 1.46). Bidirectional HMBC and COSY correlations in addition to examination of the ^13^C NMR spectrum extended the methylene chain to a terminal methyl (H_3_-34, *δ*_H_ 0.87, *t*, *J* = 7.2 Hz). The ^13^C NMR spectrum showed four nearly chemically equivalent carbon signals (C-28 and C-29, *δ*_C_ 28.9; C-30 and C-31 *δ*_C_ 28.7) correlated by HSQC to a methylene envelope (*δ*_H_ 1.22–1.25). While the overlapping methylenes made NMR correlations of individual signals difficult, the chemical shifts of these four remaining CH_2_ groups and the molecular formula of **3** supported assigning this final residue as 3-hydroxydodecanoic acid (3-Hdda) (Table 2).

The individual residue spin systems of **3** were connected using HMBC data. An HMBC correlation between the NH of Phe (NH-1, *δ*_H_ 7.40) and C-10 (*δ*_C_ 168.2) of Gly-1 connected these two residues. An HMBC correlation between the NH proton (NH-2, *δ*_H_ 8.02) and C-12 (*δ*_C_ 171.3) connected Gly-1 to Tyr. The NH proton of Tyr (NH-3, *δ*_H_ 8.98) showed an HMBC correlation to the carbonyl of Gly-2 (C-21, *δ*_C_ 172.4) and the NH (NH-4, *δ*_H_ 8.61) of Gly-2 showed an HMBC correlation to C-23 (*δ*_C_ 170.7) of the 3-Hdda residue. An HMBC correlation between the oxymethine H-25 (*δ*_H_ 5.14) and C-1 (*δ*_C_ 170.1) connected the fatty acid residue to the Phe residue, satisfied the final degree of unsaturation and established **3** as a cyclic depsipeptide featuring a 3-hydroxy fatty acid moiety. Thus, the planar structure of **3** was determined to be [3-Hdda-Gly-Tyr-Gly-Phe].

The absolute configuration of the *α*-amino acids in **3** were determined using the Marfey’s protocol described above. The hydrolyzate of **3** showed retention times (min) that matched l-Phe (13.56) and d-Tyr (11.93; see Experimental Section and Figure S24).

The absolute configuration of C-25 in the 3-Hdda residue was determined using a modified Mosher’s esterification procedure [28] on a hydrolyzed linear derivative of unnarmicin D (**4**) (Figures S25 and S26). Positive Δ(*δ*_H_*S*-*δ*_H_*R*) values for H-27 and H-26 and negative Δ(*δ*_H_*S*-*δ*_H_*R*) values for H-24 and NH-4 supported an 25*R* configuration (Figure S27).

### 2.2. Biological Evaluation of ***1**–**3***

No antibacterial activity was observed for **1**–**3** against the pathogenic strains used in this study and **3** did not demonstrate a 50% reduction in cell viability against HCT-116 cells and Neuro-2A cells (25 μM dose). Tricholides A (**1**) and B (**2**) did not show cytotoxic effects against HCT-116 cells at 25 μM. However, **2** did show moderately potent cytotoxicity against Neuro-2A neuroblastoma cells (EC_50_: 14.5 ± 6.2 μM) (See Figure S28). **1** was not tested against Neuro-2A cells to preserve some amount as a chemical standard. Further work will explore biological activities of **1**–**3** and search for additional analogs from future *Trichodesmium* collections.

## 3. Discussion

Tricholides A and B (**1** and **2**) represent structurally intriguing new additions to macrocylic PKS-NRPS molecules isolated from cyanobacteria collections. These molecules feature a core 15-membered macrolactone reminiscent of palmyrolide A [10] and the laingolides [29]. However, the tricholides feature a 2-methylhexanoic moiety instead of an unusual *t*-butyl branch. Using Tanimoto scoring and database searching, desulfated penarolide sulfate A1 was the most similar molecule to the tricholides [30]. Penarolide A1 is a macrocylic polyketide featuring a single proline residue. However, the core structure of penarolide A1 is a much larger 30-membered ring.

Unnarmicin D (**3**) showed significant structural similarity to the unnarmicins. **3** contains two chiral amino acids, while the unnarmicins contains four chiral amino acids and **3** contains a longer *β*-acyloxy chain than the unnarmicins. When comparing the amino acid and hydroxy acid sequences of five residue depsipeptides with *β*-acyloxy functionalities a pattern emerges with respect to the absolute configuration of the individual residues (Table 3). The acyloxy group is of the *R* configuration in all molecules in Table 3 except for turnagainolide B, which contains a rare 3-hydroxy-5-phenyl-4-pentenoic acid in the *S* configuration [20]. Following a predicted biosynthetic route, the first amino acid in five-residue depsipeptides with *β*-acyloxy components is in the l-configuration, the second amino acid is in the d-configuration. The third amino acid is in the l- or d-configuration and the final amino acid is in the l-configuration. Configuration analysis of new metabolites in this class, and determining the absolute configuration of unassigned compounds such as the ngercheumicins, will determine if this pattern, with respect to absolute configurations, continues to hold true.

The configurations of the acyloxy residue listed in all Table 3 examples were determined using the Mosher’s method. A computational approach was employed in the configuration analysis of the depsipeptide kailuin B, following equivocal results from derivative analysis using Mosher’s method [24]. Theodore et al., used the ^13^C NMR chemical shifts of a diagnostic set of depsipeptides containing *β*-acyloxy groups to provide key insights into ^13^C NMR *δ* values for configuration analysis. These ^13^C NMR values from experimental approaches were combined with computations using density functional theory (DFT) calculations to identify key differences in the *β* and *γ* positions of the *β*-acyloxy residue. A diagnostic assessment was proposed in which an *R* configuration is assigned when *δ*_C_*β* ≤ 74/*δ*_C_*γ* ≤ 36 while an *S* configuration is assigned when *δ*_C_*β* ≥ 77/*δ*_C_*γ* ≥ 40. The ^13^C NMR values for the *β* and *γ* positions of **3** are 72.5 ppm and 33.8 ppm respectively. Analysis of the Mosher’s ester derivatives of **4** was challenging due to overlapping methylene signals in the Hdda residue and we relied on differences in chemical shift values at H-27, H-26, H-24 and NH-4 for absolute configuration determination. There was no apparent chemical shift difference at H-25. Utilizing the diagnostic approach of Theodore et al., supports the value of these computational approaches and additionally serves as an orthogonal approach to derivitization analysis.

## 4. Materials and Methods

### 4.1. General Experimental Procedures

Optical rotations were measured using a Jasco P-2000 polarimeter (Jasco Inc., Easton, MD, USA). UV spectra were measured using a Beckman Coulter DU-800 spectrophotometer (Beckman Coulter Inc., Brea, CA, USA). NMR spectra were collected using a Bruker 800 MHz NMR instrument (Bruker, Rheinstetten, Germany) equipped with a cryoprobe with CDCl_3_ and DMSO-*d*_6_ as the internal standard (*δ*_C_ 77.0, *δ*_H_ 7.26; *δ*_C_ 39.5, *δ*_H_ 2.50). A Varian 500 MHz NMR spectrometer equipped with a 5 mm, room temperature OneNMR probe was utilized for certain experiments (Varian Inc., Palo Alto, CA, USA). HRESIMS analysis was performed using a AB SCIEX TripleTOF 4600 mass spectrometer (SCIEX, Framingham, MA, USA) with Analyst TF software. Semi-preparative HPLC was carried out using a Dionex Ultimate 3000 HPLC system equipped with a micro vacuum degasser, an autosampler and a diode–array detector (Thermo Scientific, Waltham, MA, USA).

### 4.2. Collection of Biological Material

Samples from a localized bloom of *Trichodesmium thiebautii* were collected from Padre Island, Corpus Christi, TX during 9–11 May 2014. Surface bloom material was collected in 5-g buckets from ca. 0.5-m water depth. Approximately 300 g wet weight cell mass was concentrated from this material and frozen for further chemical analysis. In the laboratory, a subsample of the cell mass was examined microscopically and identified using Komarek (2002) [31].

### 4.3. Extraction and Isolation

The frozen biomass was thawed and extracted five times using 2:1 CH_2_Cl_2_/CH_3_OH, affording 3.95 g of crude extract. The crude extract was further fractionated over silica gel using vacuum liquid chromatography (VLC) and a solvent system of increasing polarity using a stepped gradient from 100% hexanes to 100% CH_3_OH to generate 9 subfractions (A-I). Two fractions were combined (40% hexanes in ethyl acetate (Fraction E) and 20% hexanes in ethyl acetate (Fraction F)) based on similarities in their respective ^1^H NMR spectra. The combined fraction was evaporated under reduced pressure and the resultant residue was applied to a 2 g C18 SPE column and eluted with 100% methanol to remove exceedingly lipophilic constituents before HPLC separation. The fraction was subjected to RP semi-preparative HPLC using a YMC 5 μm ODS column (250 × 10 mm); mobile phase: 85% CH_3_CN/15% H_2_O with 0.05% formic acid added to each solvent, flow 3 mL/min and 0.3 mg of **1** (*t*_R_, 12.8 min) and 0.8 mg of **2** (*t*_R_, 14.0 min) were isolated. Two addition fractions, the first eluting with 100% EtOAc (fraction G) and the second eluting with 25% CH_3_OH in EtOAc (fraction H) were combined based on similarities in their ^1^H NMR spectra and dried using rotary evaporation. The residue was applied to a 2 g C18 SPE column and fractionated using 50% CH_3_CN in H_2_O, 100% CH_3_CN and 100% CH_3_OH. The fraction eluting with 100% CH_3_CN was subjected to RP semi-preparative HPLC using a YMC 5 μm ODS column (250 × 10 mm); mobile phase: 70% CH_3_CN/30% H_2_O with 0.05% formic acid added to each solvent, flow 3 mL/min and a fraction was collected from min 9.5–10.0. This fraction was further purfied using a YMC 5 μm ODS column; mobile phase: 60% CH_3_CN/40% H_2_O with 0.05% formic acid added to each solvent, flow 3 mL/min and 3.5 mg of **3** were isolated (*t*_R_, 19.0 min).

*Tricholide A* (**1**): colorless oil; ${\left[\alpha \right]}_{D}^{23}$ −10.4 (MeOH, *c* 0.09); UV (MeOH) *λ*max (log *ε*) 213 (3.9) nm ^1^H NMR (800 MHz, CDCl_3_) and ^13^C NMR (200 MHz, CDCl_3_) see Table 1; HRESIMS *m*/*z* 408.3113 [M + H]^+^ (calcd. for C_24_H_42_NO_4_, 408.3114).

*Tricholide B* (**2**): colorless oil; ${\left[\alpha \right]}_{D}^{23}$ −10.8 (MeOH, *c* 0.09); UV (MeOH) *λ*max (log *ε*) 205 (3.5) nm ^1^H NMR (800 MHz, CDCl_3_) and ^13^C NMR (200 MHz, CDCl_3_), see Table S1; HRESIMS *m*/*z* 422.3276 [M + H]^+^ (calcd. for C_25_H_44_NO_4_, 422.3270).

*Unnarmicin D* (**3**): white amorphous solids; ${\left[\alpha \right]}_{D}^{22}$ −53.3 (MeOH, *c* 0.07); UV (MeOH) *λ*max (log *ε*) 201 (4.09), 221 (3.76), 275 (3.02) nm; ^1^H NMR (800 MHz, DMSO-*d*_6_) and ^13^C NMR (200 MHz, DMSO-*d*_6_), see Table 2; HRESIMS *m*/*z* 623.3436 [M + H]^+^ (calcd. for C_34_H_47_N_4_O_7_, 623.3445).

### 4.4. Acid Hydrolysis and Marfey’s Protocol

To determine the absolute configuration of the *α*-amino acids in **1**–**3**, 0.2 mg of each compound was reconstituted in 0.2 mL of 6 N HCl and heated at 110 °C for 15 h. The hydrolyzate was dried in vacuo, reconstituted in 100 µL of 0.1 M NaHCO_3_ solution and treated with 50 µL of 1 mg/mL solution of *N*-*α*-(2,4-dinitro-5-fluorophenyl)-l-valinamide (L-FDVA) in acetone, followed by heating at 55 °C for 2 h, cooled to room temperature and quenched with 50 µL of 2 N HCl. The hydrolyzate was dried in vacuo and reconstituted in 100 µL of 50% CH_3_CN/H_2_O + 0.1% formic acid (FA) and filtered through 0.2 µm filter. The hydrolyzate and the L-FDVA derivatized *α*-amino acid standards were subjected to HPLC analysis (20% CH_3_CN/H_2_O + 0.1% FA to 80% CH_3_CN/H_2_O + 0.1% FA over 30 min; Phenomenex Kinetex C18 column, 150 × 3 mm, flow 0.6 mL/min). The retention time (min) of the hydrolyzate of **1** and **2** matched l-Pro (13.00; d-Pro, 14.50). The retention times (min) of the hydrolyzate of **3** matched l-Phe (13.55; d-Phe, 16.18), and d-Tyr (11.75; l-Tyr, 10.45).

### 4.5. Hydrolysis of ***3***

Unnarmicin D (**3**) (3.0 mg) was dissolved in 5% sodium methoxide in CH_3_OH (2 mL) and stirred for 30 min at room temperature. The reaction mixture was neutralized by adding 100 μL of 1 M HCl. The mixture was evaporated under reduced pressure to remove CH_3_OH and 1 mL of H_2_O was added to the slurry and extracted three times with EtOAc. After drying with over Na_2_SO_4_, the solvent was removed under reduced pressure and the residue was filtered and subjected to RP-HPLC using a YMC 5 μm ODS column (250 × 10 mm); mobile phase: 65% CH_3_CN/35% H_2_O with 0.05% formic acid added to each solvent, flow 3 mL/min and 0.50 mg of **4** was isolated (*t*_R_, 7.0 min).

*Unnarmicin D linear derivative* (**4**): white amorphous solids; ^1^H NMR (500 MHz, DMSO-*d*_6_) *δ* 0.85 (3H, t, *J* = 7.2 Hz, Hdda, H-34), 1.16 (2H, m, Hdda, H-27) 1.21–1.26 (12H, ovlp, Hdda, H-28-H-33), 1.33 (2H, m, Hdda, H-26), 2.20 (2H, m, Hdda, H-24), 2.63 (1H, m, Tyr, H-14b), 2.85 (1H, m, Tyr, H-14a), 2.87 (1H, m, Phe, H-3b), 3.03 (1H, m, Phe, H-3a), 3.39 (1H, m, Gly-1, H-11b), 3.47 (1H, m, Gly-2, H-22b), 3.59 (1H, m, Gly-2, H-22a), 3.75 (1H, m, Hdda, H-25), 3.80 (1H, m, Gly-1, H-11a), 4.34 (1H, m, Tyr, H-13) 6.61 (2H, d, *J* = 8.5 Hz, Tyr, H-17/19), 6.96 (2H, d, *J* = 8.5 Hz, H-16/20) 7.11 (3H, ovlp, Phe, H-5/9, H-7), 7.15 (2H, m, Phe, H-6/8) 7.32 (1H, m, NH-1), 7.95 (1H, m, NH-3), 8.20 (1H, m, NH-2), 8.35 (1H, m, NH-4); HRESIMS *m*/*z* 663.3366 [M + Na]^+^ (calcd. for C_34_H_48_N_4_O_8_, 663.3370).

### 4.6. Preparation of MTPA Esters of ***4***

0.25 mg of **4** was dissolved in dry CH_2_Cl_2_ (0.6 mL) in a 4 mL vial to which dry pyridine (10 µL) and (*S*)-(+)-*α*-methoxy-*α*-(trifluoromethyl)phenylacetyl chloride (15 µL) were added. The identical procedure was repeated with an equal amount of **4** and (*R*)-(−)-*α*-methoxy-*α*-(trifluoromethyl)phenylacetyl chloride. The vials were capped and the reaction mixtures were stirred for 24 h. The reactions were quenched with H_2_O and separated using CH_2_Cl_2_ and H_2_O. The CH_2_Cl_2_ layer was evaporated under reduced pressure and the derivatives were subjected to ^1^H NMR analysis.

### 4.7. Antimicrobial Assay

#### 4.7.1. Bacterial Strains

Methicillin-resistant *Staphylococcus aureus* (MRSA, ATCC 43300), *Pseudomonas aeruginosa* PAO1, and Escherichia coli (ATCC 35218) were employed for antimicrobial activities.

#### 4.7.2. Antimicrobial Activity

The antimicrobial properties of **1**–**3** were tested via broth dilution assay. Compounds were prepared in DMSO at 5 mg/mL. Tetracycline and gentamycin were used as positive controls. Minimal inhibitory concentrations (MICs) were determined using the broth microdilution method. Briefly, pathogens were firstly inoculated into tryptic soy broth (TSB, BD, Franklin Lakes, NJ, USA) at 37 °C and shaken at 175 rpm overnight. The bacterial broth (1 × 10^8^ colony-forming units (CFU)/mL) was immediately diluted to 1 × 10^5^ CFU/mL. In 96-well microtiter plates, 5 μL of compounds and controls were mixed well with 195 μL of bacterial suspension (1:39 (*v*/*v*)). After a series of two-fold dilutions, the microtiter plates were then incubated statically at 37 °C overnight. MICs were determined as the minimal concentration at which no visible bacterial growth was present. No antimicrobial activity was observed for compounds **1**–**3**.

### 4.8. Cytotoxicity Assay

HCT-116 cells and Neuro-2A cells were added to 96 well plates in 100 µL of McCoy’s Media and Eagle’s Minimum Essential Media (EMEM) respectively each supplemented with 10% FBS each at a density of 5000 cells/well. Cells were incubated overnight (37 °C, 5% CO_2_) and examined microscopically to confirm confluence and adherence. Purified **1**–**3** were dissolved in DMSO (1% *v*/*v*) and added to the cells in the range of 100, 10, 1, 0.1 and 0.01 µM. Four technical replicates were prepared for each concentration and the assay was performed in triplicate. Doxorubicin was used as the positive control (EC_50_:Neuro-2A = 100 ± 5.2 nM). Plates were incubated for 72 h after which 15 µL of MTT dye were added each assay well. The dye was allowed to incubate with the cells for 4 h after which the media was aspirated and the remaining crystals were solubilized in 100 µL DMSO. The plates were incubated at 37 °C for 30 min to allow the crystals to solubilize. Absorbance at 540 nm was measured using a Molecular Devices SpectraMax plate reader and % viability was calculated compared to the negative control (1% DMSO) and EC_50_ curves were generated using GraphPad Prism 6.

## Figures and Tables

**Table 1:** NMR data for tricholide A (**1**) ^a^ (CDCl_3_).

Position	*δ*_C_	*δ*_H_ (*J* in Hz)	HMBC	COSY
1	172.2, qC			
2	59.1, CH	4.50, dd (9.0, 2.0)	1, 3, 4, 5	3a, 3b
3a	31.8, CH_2_	2.29, m	1, 2, 4	2, 3b, 4
3b		2.10, m	1, 4, 5	3a, 4
4	22.3, CH_2_	1.94, m	2, 3, 5	3a, 5a, 5b
5a	46.7, CH_2_	3.77, m	2, 3, 4, 6	4, 5b
5b		3.62, m	2, 3, 4, 6	4, 5a
6	165.2, qC			
7	117.1, CH	5.84, d (15.5)	6, 8, 9, 24	8
8	151.1, CH	7.16, dd (15.5, 5.8)	6, 7, 9, 10, 24	7, 9
9	35.9, CH	2.34, m	7, 8, 10, 24	8, 10, 24
10	30.3, CH_2_	1.36, m	8, 9, 11	9
11a	30.9, CH_2_	1.62, m	10, 12	11b, 12
11b		1.43, ovlp ^b^	10, 12	12
12	79.9, CH	3.20, m	11, 13, 14, 23	11b, 13a
13a	30.0, CH_2_	1.55, m	12, 14	12, 13b, 14b
13b		1.43, ovlp	12, 14	12, 13a, 14b
14a	19.6, CH_2_	1.40, m	13, 15, 16	13b, 14a
14b		1.16, m	15, 16	13a, 14a
15a	32.7, CH_2_	1.57, m	14, 16	14b, 15b, 16
15b		1.47, m	14, 16	14b, 15a, 16
16	78.3, CH	4.98, ddd (10.6, 4.6)	1, 14, 15, 17, 22	15a, 15b, 17
17	37.5, CH	1.60, m	16, 18, 19, 22	16, 18b, 22
18a	32.9, CH_2_	1.34, ovlp	17, 19	17, 18b
18b		1.10, m	17, 19	17, 18a
19a	29.2, CH_2_	1.30, ovlp	18, 20	
19b		1.26, ovlp	18, 20	18b
20	22.9, CH_2_	1.28, ovlp	21	21
21	14.0, CH_3_	0.89, t (7.1)	19, 20	20
22	14.7, CH_3_	0.91, d (6.8)	16, 17, 18	17
23	56.0, CH_3_	3.27, s	12	
24	22.5, CH_3_	1.04, d (7.0)	8, 9, 10	9

^a^ 800 MHz for ^1^H, 200 MHz for ^13^C; ^b^ overlapping signals.

**Table 2:** NMR data for unnarmicin D (**3**) ^a^ (DMSO-*d*_6_).

Residue	Position	*δ*_C_	Type *δ*_H_ (*J* in Hz)	HMBC	COSY
Phe	1	170.1, qC			
	2	52.6 CH	4.58, td (9.5, 5.0)	1, 3, 4, 10	3a, 3b, NH-1
	3a	36.5, CH_2_	3.15, dd (13.7, 5.0)	1, 2, 4, 5, 9	2, 3b
	3b		2.78, ovlp	1, 2, 4, 5, 9	2, 3a
	4	137.6, qC			
	5/9	129.3, CH	7.20, d (7.2)	3, 7	
	6/8	128.0, CH	7.24, t (7.2)	4, 5, 9	
	7	126.2, CH	7.17, t (7.2)	5, 9	
	NH-1		7.40, d (9.3)	2, 10	2
Gly-1	10	168.2, qC			
	11a	42.1, CH_2_	3.90, dd (17.1, 8.3)	10, 12	11b, NH-2
	11b		3.37, dd (17.1, 4.6)	10, 12	11a, NH-2
	NH-2		8.02, (8.4, 4.8)	11, 12	11a, 11b
Tyr	12	171.3, qC			
	13	57.2, CH	4.13, m	12, 14, 15, 21	14a, 14b, NH-3
	14a	35.0, CH_2_	3.02, dd (14.3, 3.8)	12, 13, 15, 16, 20	13, 14b
	14b		2.78, ovlp	12, 13, 15, 16, 20	13, 14a
	15	127.8, qC			
	16/20	129.8, CH	7.13, d (8.5)	14, 18	17/19
	17/19	115.1, CH	6.69, d (8.5)	15, 18	16/20
	18	156.0, qC			
	NH-3		8.98, d (5.5)	13, 14, 21	13
Gly-2	21	172.4, qC			
	22a	42.7 CH_2_	3.83, dd (14.7, 3.8)	21, 23	22b, NH-4
	22b		3.51, dd (14.7, 6.8)	21, 23	22a, NH-4
	NH-4		8.61, dd (6.8, 3.9)	22, 23	22a, 22b
Hdda ^b^	23	170.7, qC			
	24a	40.4, CH_2_	2.60, dd (13.7, 3.6)	23, 25, 26	24b, 25
	24b		2.19, dd (13.7, 10.5)	23, 25, 26	24a, 25
	25	72.5, CH	5.14, m	1, 24, 26, 27	24b, 26a, 26b
	26a	33.8, CH_2_	1.54, m	24, 25, 27, 28	25, 26b, 27
	26b		1.46, m	24, 25, 27, 28	25, 26a, 27
	27	24.4, CH_2_	1.16, m	25, 26, 28	26a, 26b
	28	28.9, CH_2_	1.22, ovlp ^c^		
	29	28.9, CH_2_	1.22, ovlp		
	30	28.7, CH_2_	1.25, ovlp		
	31	28.7, CH_2_	1.25, ovlp		
	32	31.3, CH_2_	1.24, ovlp	31, 33, 34	
	33	22.1, CH_2_	1.28, m	32, 34	34
	34	14.0, CH_3_	0.87, t (7.2)	32, 33	33

^a^ 800 MHz for ^1^H, 200 MHz for ^13^C; ^b^ 3-hydroxydodecanoic acid; ^c^ overlapping signals.

**Table 3:** Five-residue depsipeptides from marine microbes containing *β*-hydroxy acid groups.

Compound	Residue Sequence	Source
Unnarmicin D (**3**)	(*R*)-Hdda Gly d-Tyr Gly l-Phe	environmental collection of *T. thiebautii*
Unnarmicin A [17]	(*R*)-Hha ^a^ l-Leu d-Phe l-Leu l-Phe	*Photobacterium* sp. strain MBIC0648517
Unnarmicin C [17]	(*R*)-Hoa ^b^ l-Leu d-Phe l-Leu l-Phe	*Photobacterium* sp. strain MBIC0648517
Solonamide A [18]	(*R*)-Hha l-Phe d-Leu d-Ala l-Leu	*Photobacterium* sp. strain S275318
Solonamide B [18]	(*R*)-Hoa l-Phe d-Leu d-Ala l-Leu	*Photobacterium* sp. strain S275318
Arthroamide [19]	(*R*)-Hppa ^c^ l-Val d-Ala l-Val l-Val	*Arthrobacter* sp. strain PGVB119
Turnagainolide A [20]	(*R*)-Hppa l-Val d-Ala l-Ile l-Val	*Bacillus* sp. strain RJA219420
Turnagainolide B [20]	(*S*)-Hppa l-Val d-Ala l-Ile l-Val	*Bacillus* sp. strain RJA219420
Ngercheumicin C [21]	Hoa Phe Leu Leu Leu	*Photobacterium* sp.
Ngercheumicin D [21]	Hoa Phe Met Leu Leu	*Photobacterium* sp.
Ngercheumicin E [21]	Hoa Phe Phe Leu Leu	*Photobacterium* sp.

^a^ 3-hydroxy-hexanoic acid; ^b^ 3-hydroxy-octanoic acid; ^c^ 3-hydroxy-5-phenyl-4-pentenoic acid.

## Supplementary Materials

The following are available online at www.mdpi.com/1660-3397/15/7/206/s1, Table S1: NMR data for **2**; Figures S1–S21: ^1^H NMR, ^13^C NMR, HSQC, HMBC, COSY, TOCSY and NOESY spectra of **1**–**3**; Figures S22–S24: Marfey’s l-FDVA derivatives of the hydrolyzate of **1**–**3** compared to l-FDVA amino acid standards. Figures S25 and S26: ^1^H NMR and COSY of **4**. S27: Δ(*δ*_H_*S*-*δ*_H_*R*) values of *S*-MTPA and *R*-MTPA esters of **4**. Figure S28. EC_50_ curve of **2** tested against Neuro-2A cells.
