# Supplementary material for: Tricholides A and B and Unnarmicin D: New Hybrid PKS-NRPS Macrocycles Isolated from an Environmental Collection of Trichodesmium thiebautii
Source: Mar Drugs. 2017 Jun 30;15(7):206. doi: 10.3390/md15070206 (PMC5532648; doi:10.3390/md15070206)
Supplement: Supplementary file 1 [file marinedrugs-15-00206-s001.pdf]

Supplementary information

Tricholides A and B and unnarmicin D: new hybrid PKS-NRPS macrocycles isolated from an environmental collection of *Trichodesmium thiebautii*

Matthew J. Bertin, Alexandre F. Roduit, Jiadong Sun, Gabriella E. Alves, Christopher W. Via, Miguel A. Gonzalez, Paul V. Zimba and Peter D. R. Moeller

Table S1. NMR data for tricholide B (**2**).
S1. ^{1}H NMR spectrum of tricholide A (**1**) (800 MHz, $CDCl_3$).
S2. ^{13}C NMR spectrum of **1** (200 MHz, $CDCl_3$).
S3. HSQC spectrum of **1**.
S4. HMBC spectrum of **1**.
S5. COSY spectrum of **1**.
S6. TOCSY spectrum of **1**.
S7. NOESY spectrum of **1**.
S8. ^{1}H NMR spectrum of tricholide B (**2**) (800 MHz, $CDCl_3$).
S9. ^{13}C NMR spectrum of **2** (200 MHz, $CDCl_3$).
S10. HSQC spectrum of **2**.
S11. HMBC spectrum of **2**.
S12. COSY spectrum of **2**.
S13. TOCSY spectrum of **2**.
S14. NOESY spectrum of **2**.
S15. ^{1}H NMR spectrum of unnarmicin D (**3**) (800 MHz, DMSO).
S16. ^{13}C NMR spectrum of **3** (200 MHz, DMSO).
S17. HSQC spectrum of **3**.
S18. HMBC spectrum of **3**.
S19. COSY spectrum of **3**.
S20. TOCSY spectrum of **3**.
S21. NOESY spectrum of **3**.
S22. Chromatographic comparison of the L-FDVA reacted acid hydrolyzate of **1** compared to authentic amino acid standards reacted with L-FDVA.
S23. Chromatographic comparison of the L-FDVA reacted acid hydrolyzate of **2** compared to authentic amino acid standards reacted with L-FDVA.
S24. Chromatographic comparison of the L-FDVA reacted acid hydrolyzate of **3** (blue UV trace) compared to authentic amino acid standards reacted with L-FDVA (black UV trace).
S25. ^{1}H NMR spectrum of unnarmicin D linear derivative (**4**) (500 MHz, DMSO).
S26. COSY spectrum of unnarmicin D linear derivative (**4**).
S27. $\Delta(\delta_H S\text{-}\delta_H R)$ values of *S*-MTPA and *R*-MTPA esters of **4**.
S28. Cytotoxicity of **2** against Neuro-2A mouse neuroblastoma cells. The dosing regime was carried out in triplicate.

Table S1. NMR data for tricholide B (**2**) (800MHz, $CDCl_3$)

Position	δ_C	δ_H (*J* in Hz)	HMBC	COSY
1	171.7, qC			
2	60.7, CH	4.69, dd (8.5, 3.4)	1, 3, 4, 5	2a, 2b
3a	31.7, CH_2	2.38, m	1, 2, 4, 5	2, 3a, 4b
3b		2.18, m	1, 2, 4, 5	2, 3a, 4a
4a	22.7, CH_2	1.93, m	2, 3, 5	4b, 5b
4b		1.79, m	2, 3, 5	3a, 4a, 5a, 5b
5a	46.6, CH_2	3.76, m	2, 3, 4, 6	4a, 4b, 5b,
5b		3.54, m	2, 3, 4, 6	4a, 4b, 5a
6	173.0, qC			
7	133.0, qC			
8	136.1, CH	5.24, d (9.5)	6, 9, 24, 25	9
9	32.6, CH	2.43, m	7, 8, 10, 11, 24	8, 10b, 24
10a	33.2, CH_2	1.72, m	8, 9, 11, 24	10b, 11a
10b		1.10, m	8, 9, 11, 24	9, 10a
11a	30.8, CH_2	1.53, m	10, 12	10a, 11b
11b		1.27, ovlp[a]	10, 12	11a
12	80.3, CH	3.09, m	10, 13, 14, 23	11a, 11b, 13a, 13b
13a	30.9, CH_2	1.69, m	11, 12, 14	11a, 12, 13b
13b		1.30, ovlp	11, 12, 14	12, 13a
14a	19.9, CH_2	1.37, m	13, 15, 16	14b
14b		1.20, m	13, 15, 16	13a, 14a
15	30.2, CH_2	1.52, m	13, 14, 16	16
16	78.7, CH	4.81, m	1, 14, 17, 18, 22	15, 17
17	36.6, CH	1.63, m	16, 18, 19, 22	22
18a	32.2, CH_2	1.32, ovlp	16, 17, 19, 20, 22	17, 18b
18b		1.04, m	16, 17, 19, 20, 22	17, 18a
19a	29.2, CH_2	1.30, ovlp	17, 20	19b
19b		1.22, m	17, 20	19a
20a	22.9, CH_2	1.29, ovlp	18, 19, 21	14
20b		1.25, ovlp	18, 19, 21	14
21	14.0, CH_3	0.88, t (6.4)	19, 20	20b
22	15.0, CH_3	0.86, d (6.8)	16, 17, 18	17
23	56.3, CH_3	3.30, s	12	
24	21.3, CH_3	0.99, d (6.6)	8, 9, 10	9
25	14.8, CH_3	1.84, s	6, 7, 8	8

[a]overlapping signals

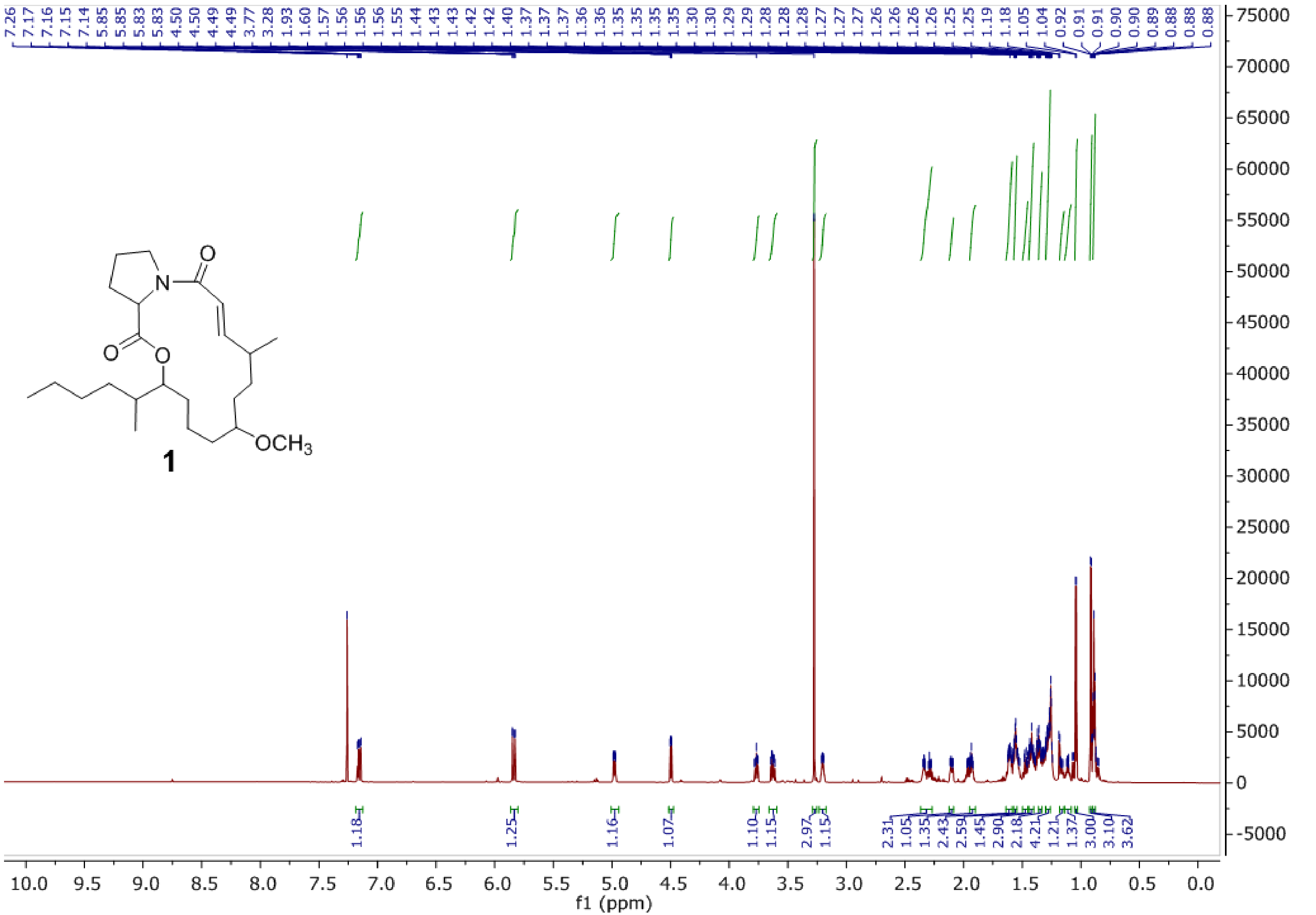

S1. ^{1}H NMR spectrum of tricholide A (**1**) (800 MHz, $CDCl_3$).

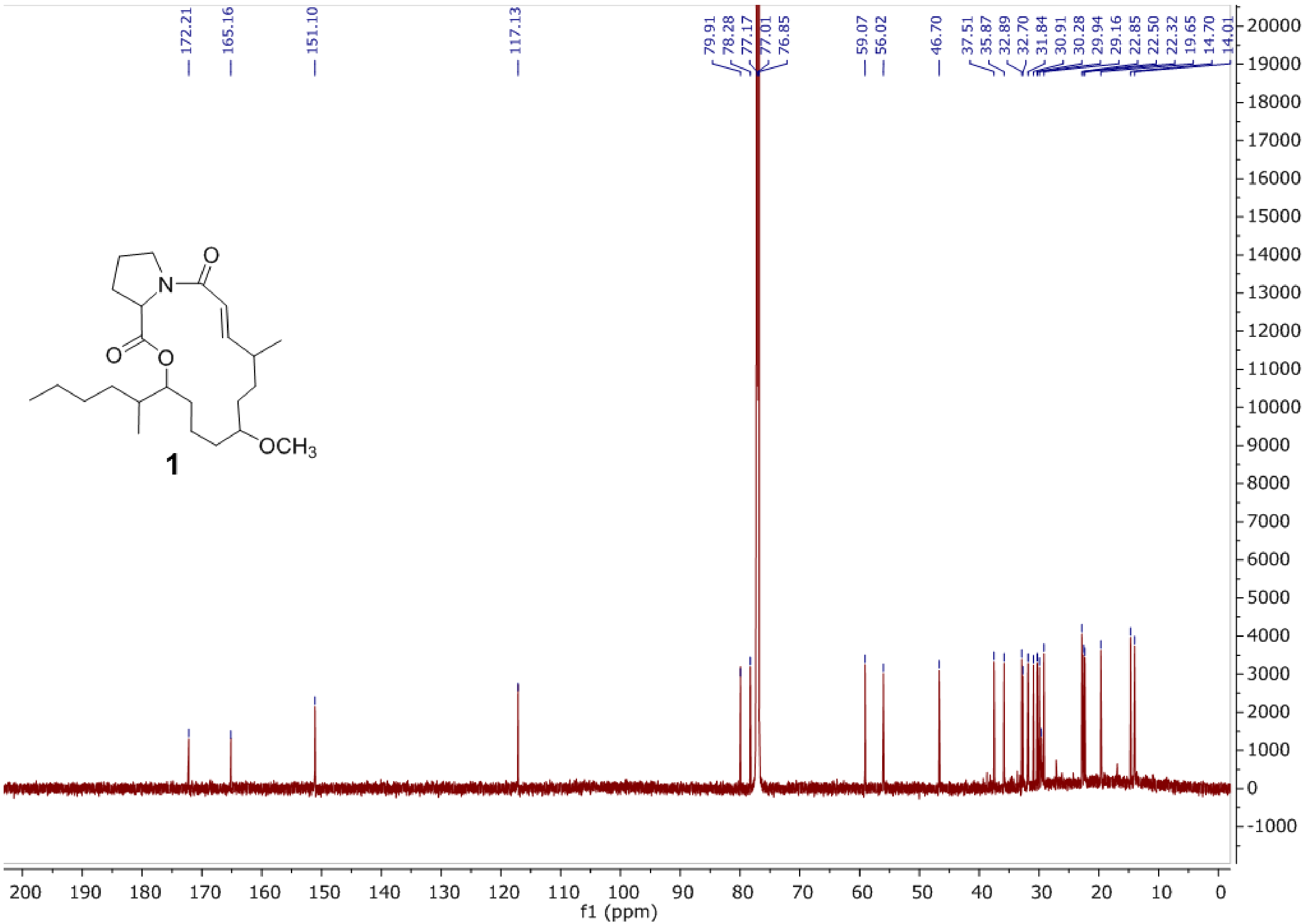

S2. ^{13}C NMR spectrum of **1** (200 MHz, $CDCl_3$).

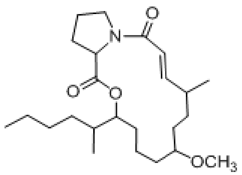

1

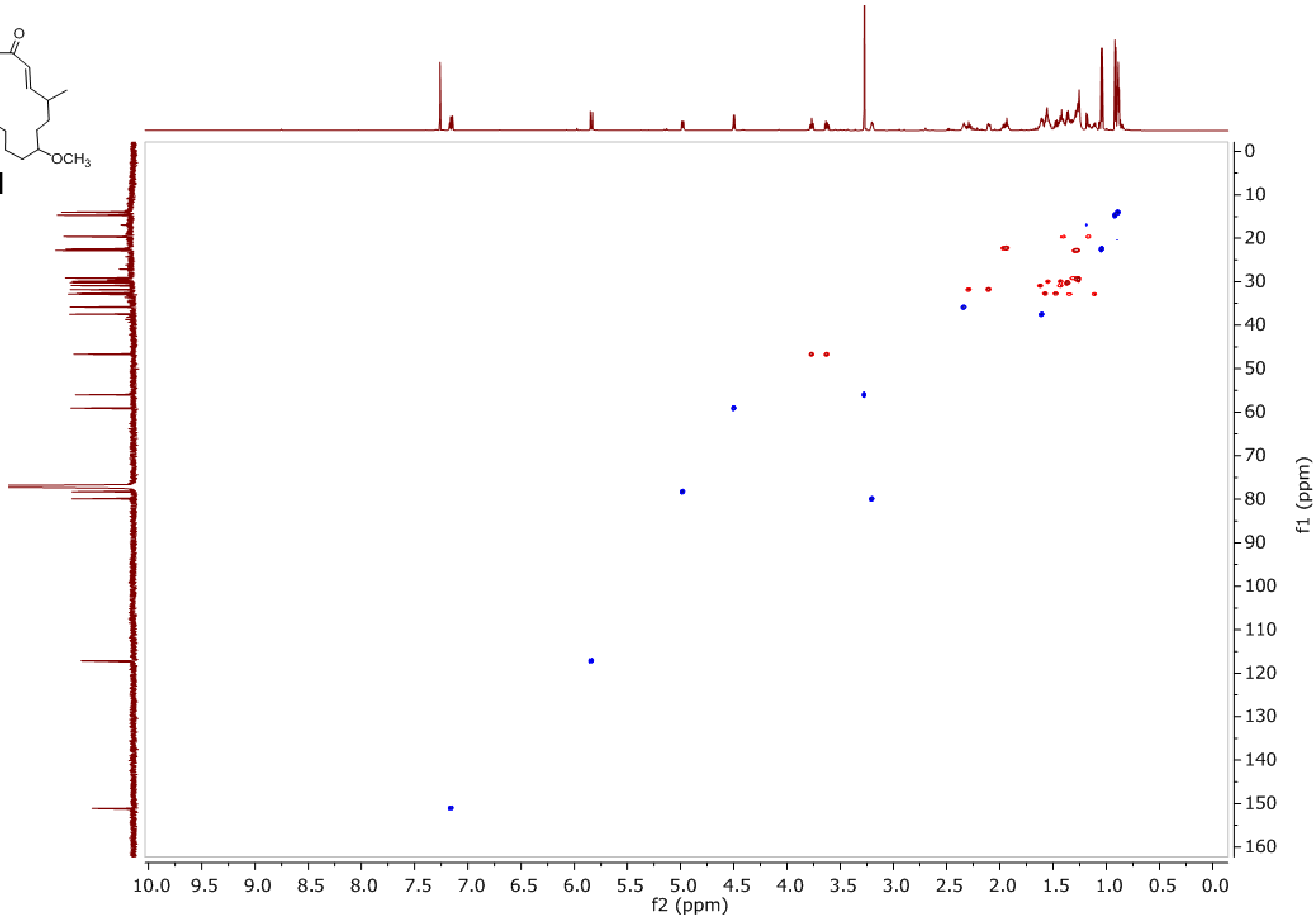

S3. HSQC spectrum of **1**.

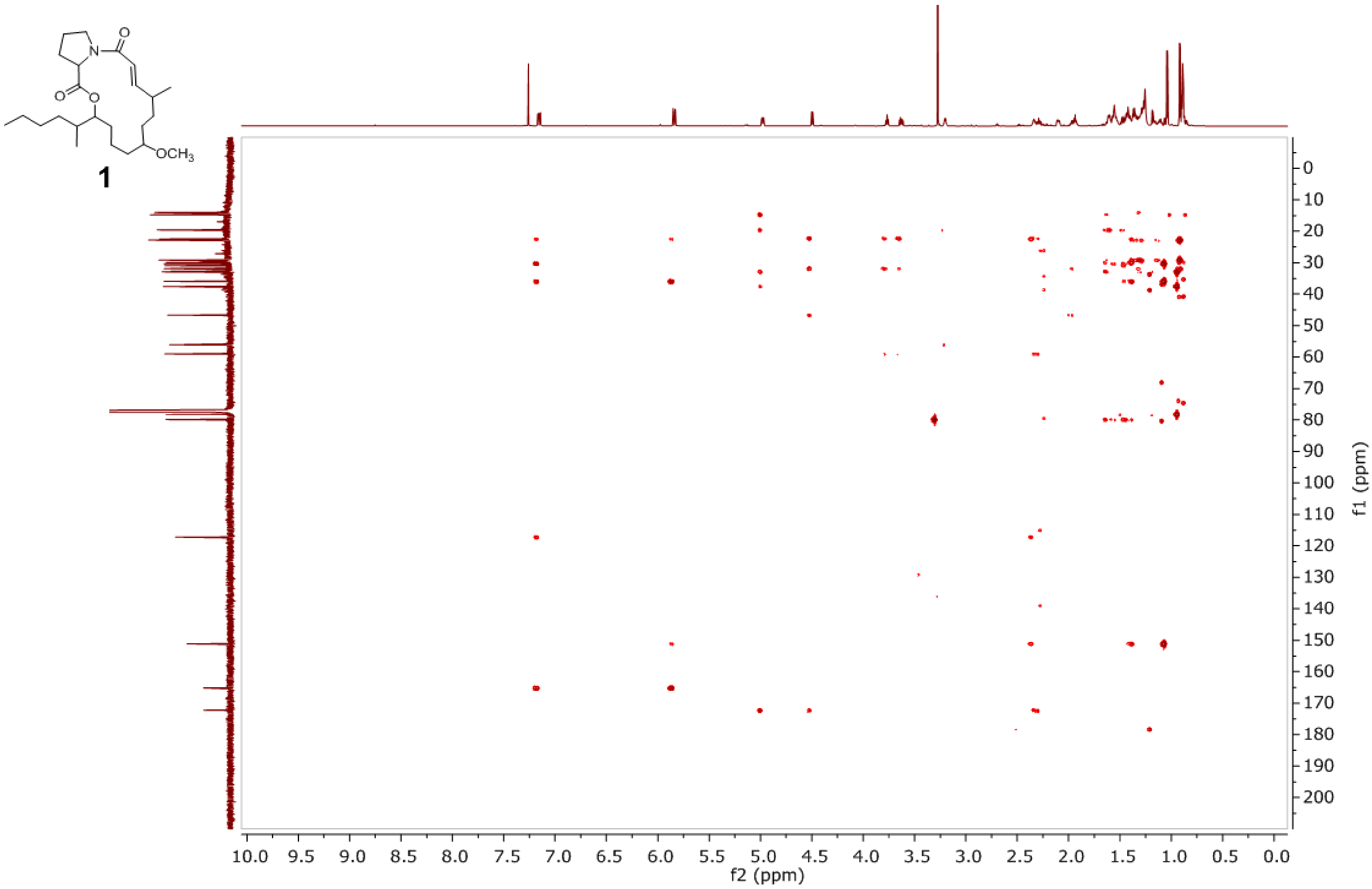

S4. HMBC spectrum of **1**.

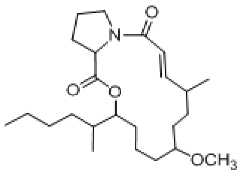

1

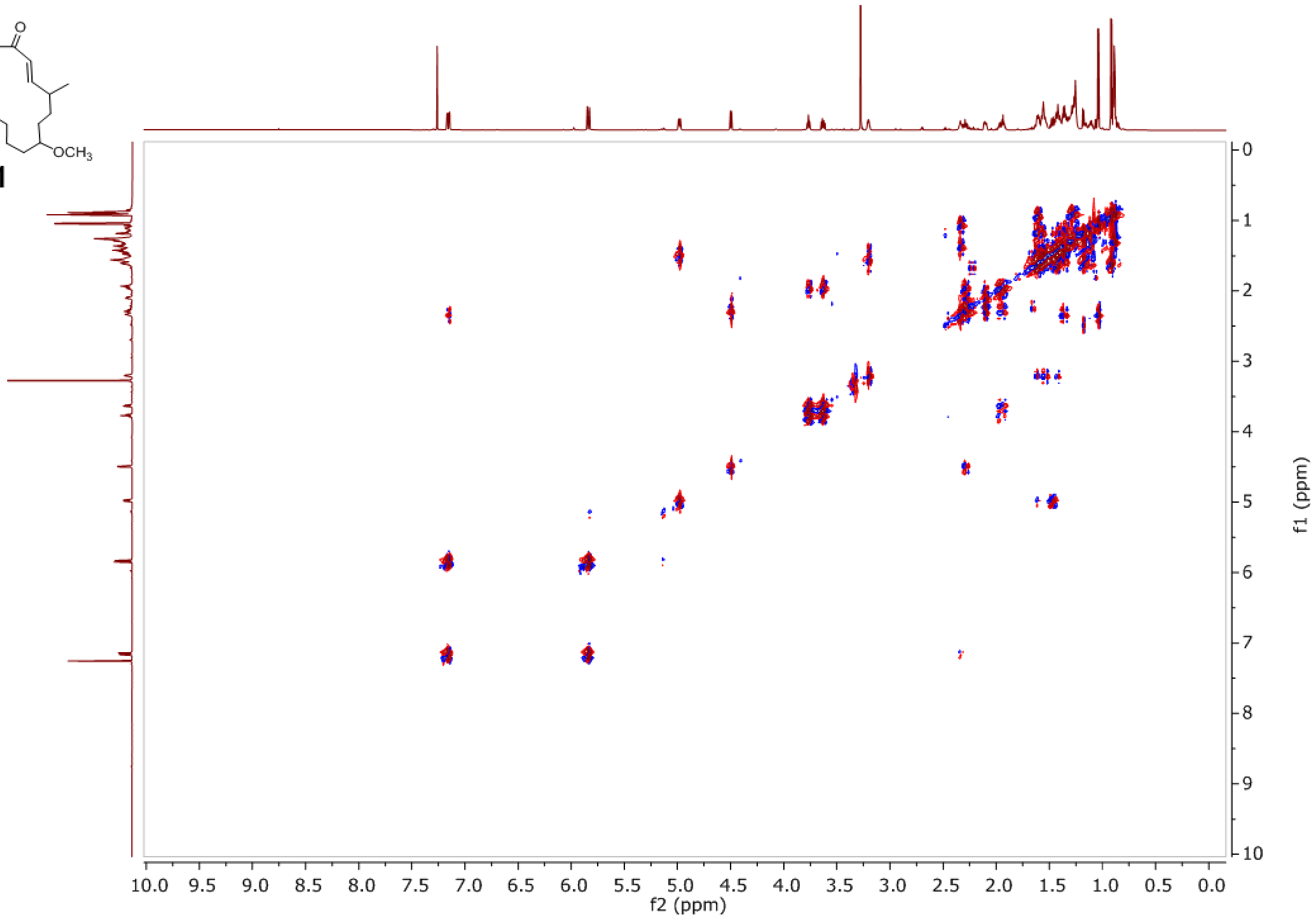

S5. COSY spectrum of **1**.

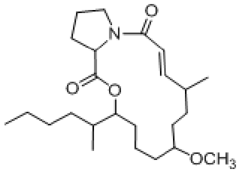

1

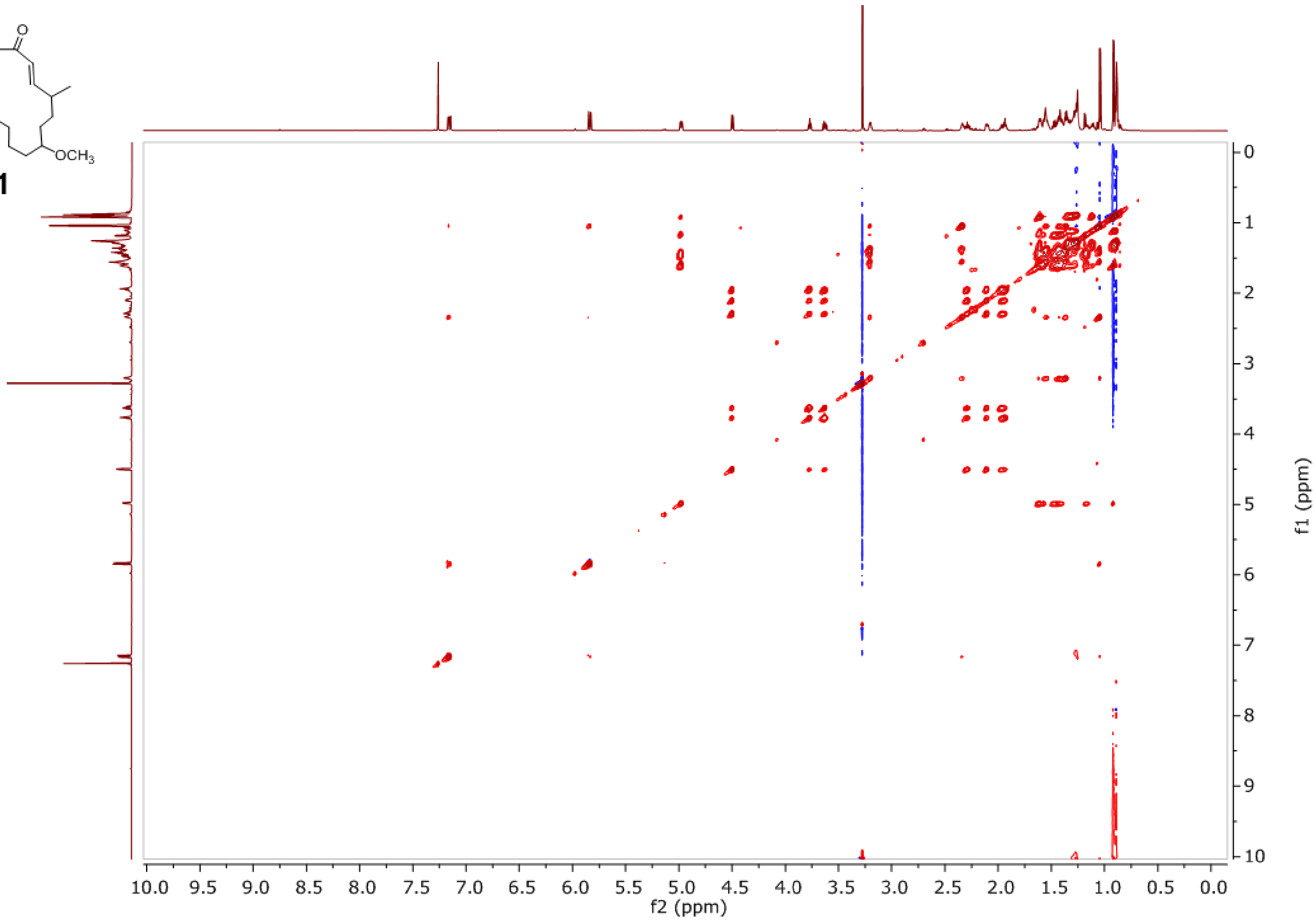

S6. TOCSY spectrum of **1**.

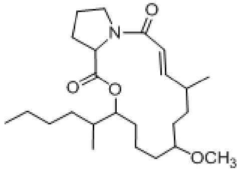

1

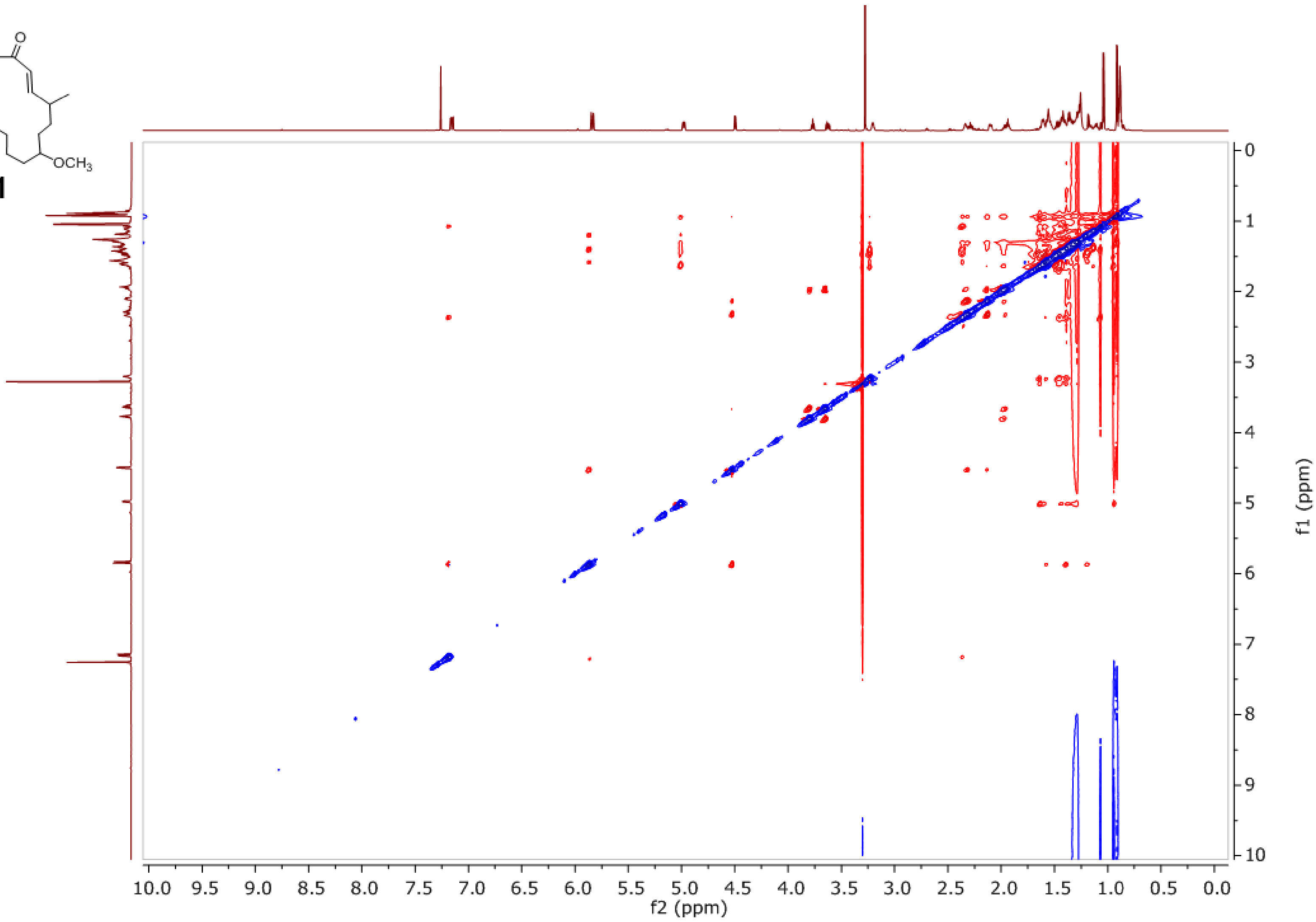

S7. NOESY spectrum of **1**.

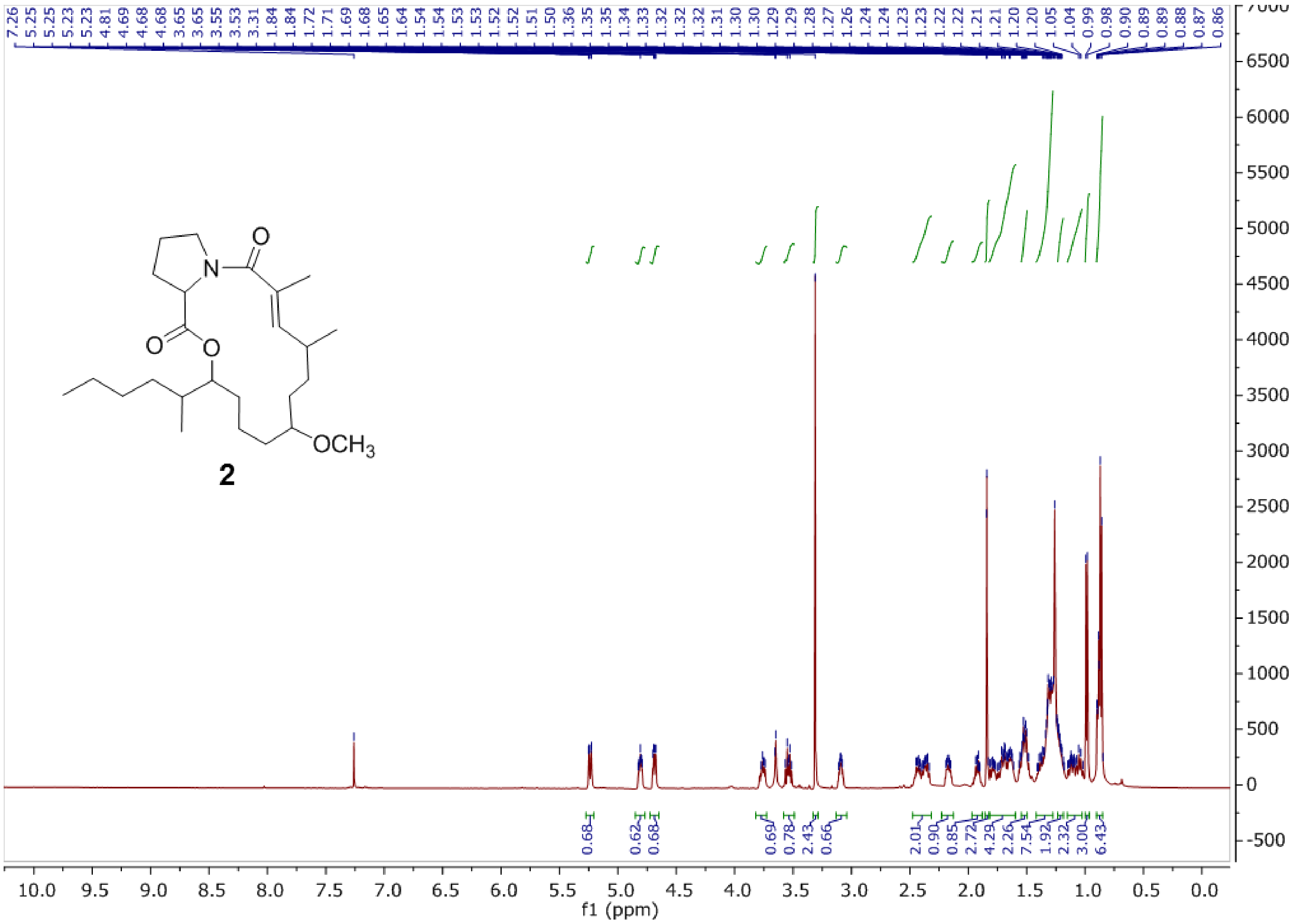

S8. ^{1}H NMR spectrum of tricholide B (**2**) (800 MHz, $CDCl_3$).

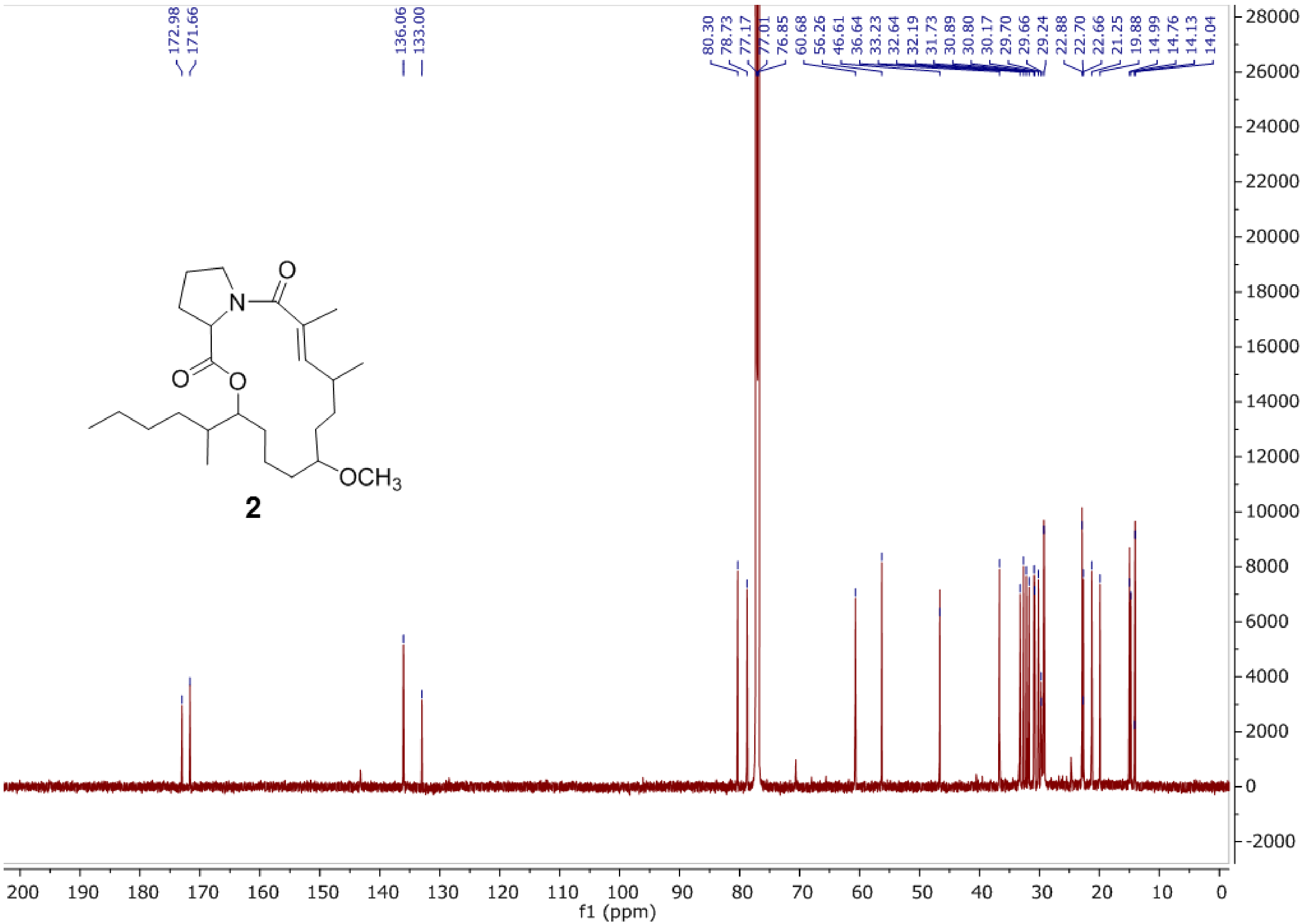

S9. ^{13}C NMR spectrum of **2** (200 MHz, $CDCl_3$).

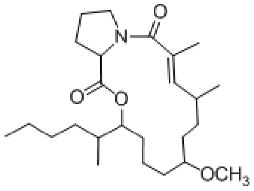

2

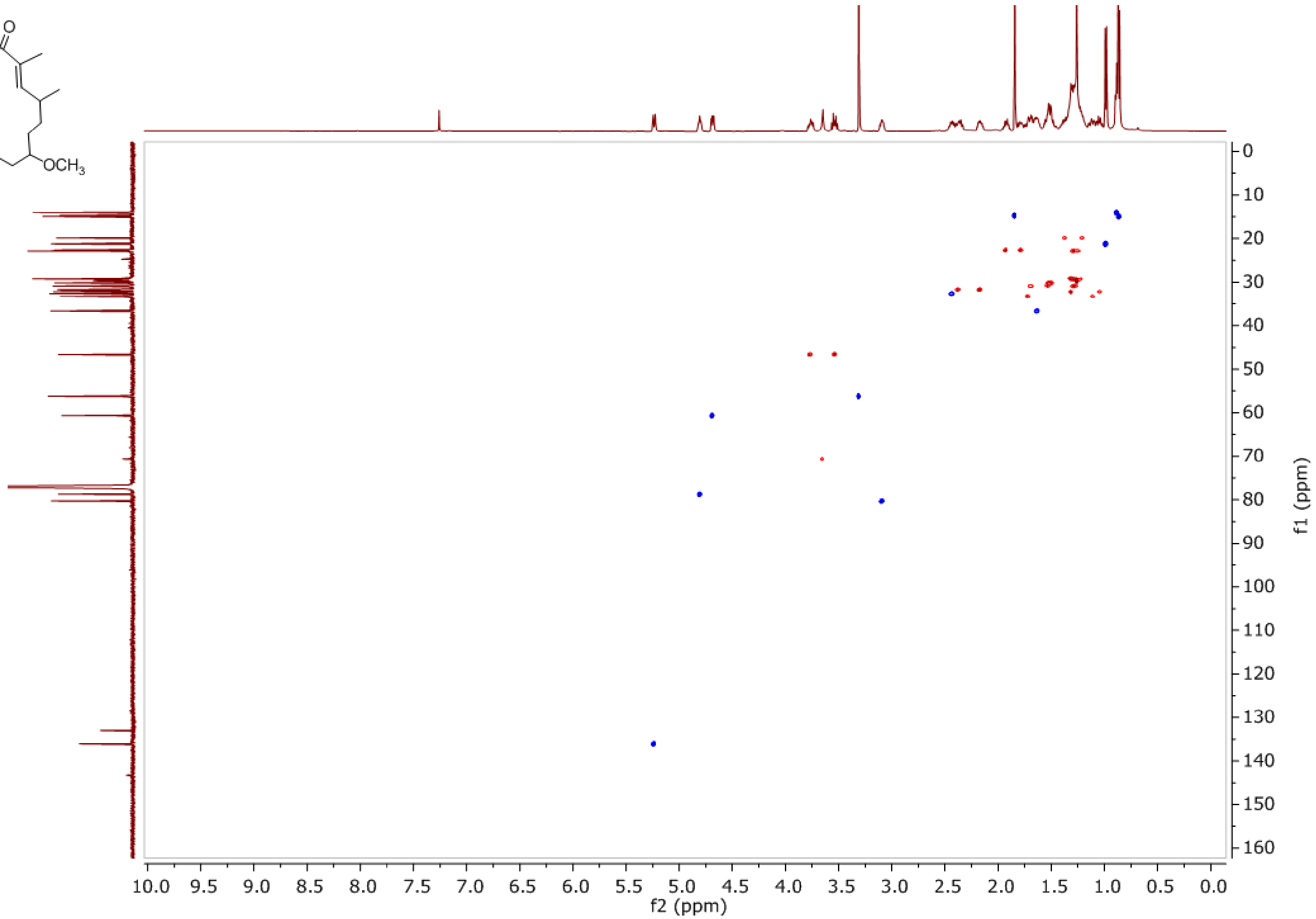

S10. HSQC spectrum of **2**.

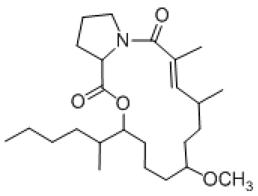

2

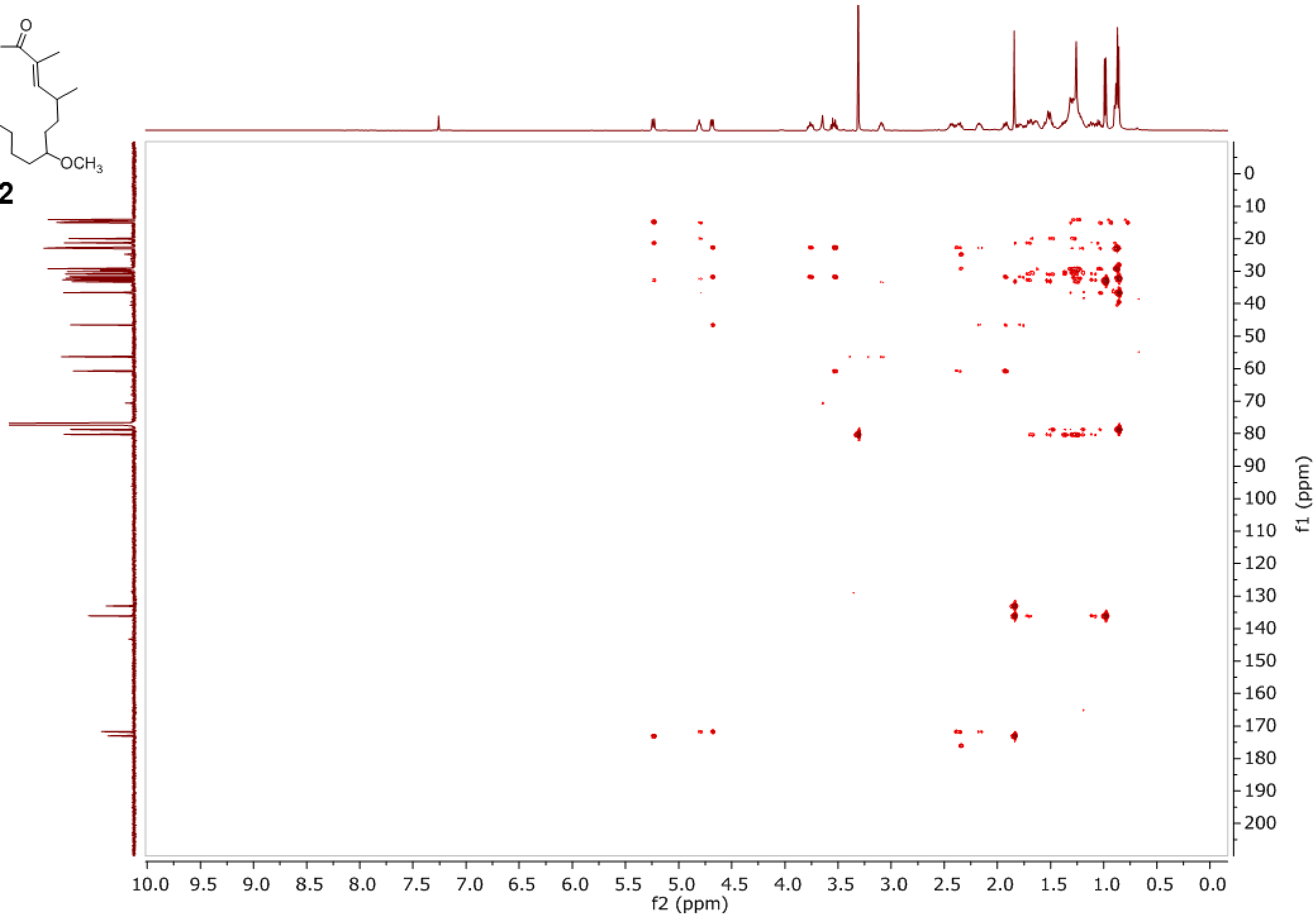

S11. HMBC spectrum of **2**.

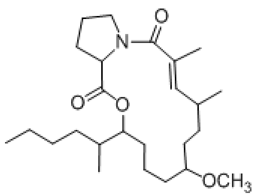

2

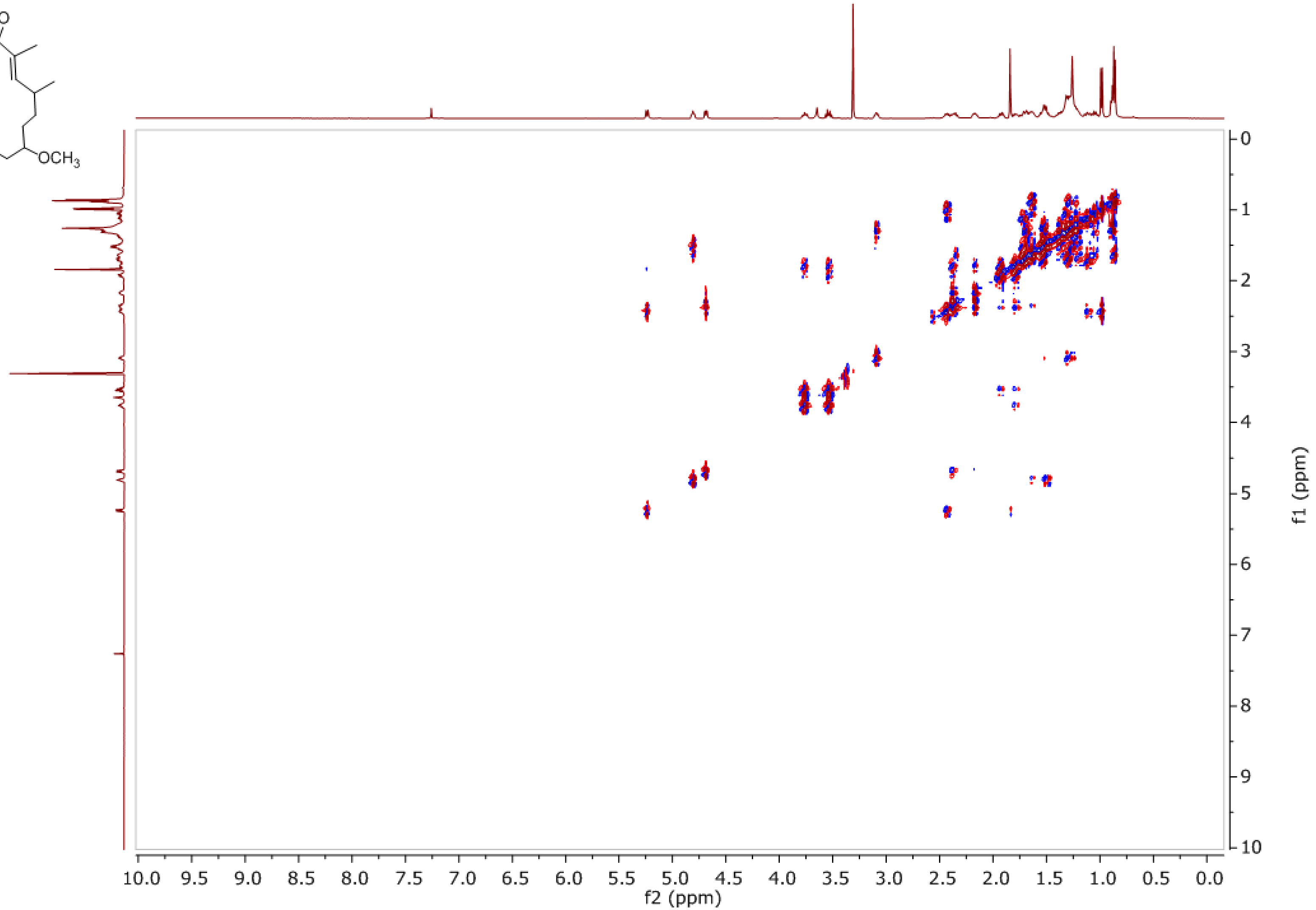

S12. COSY spectrum of **2**.

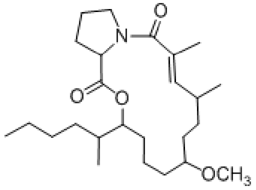

2

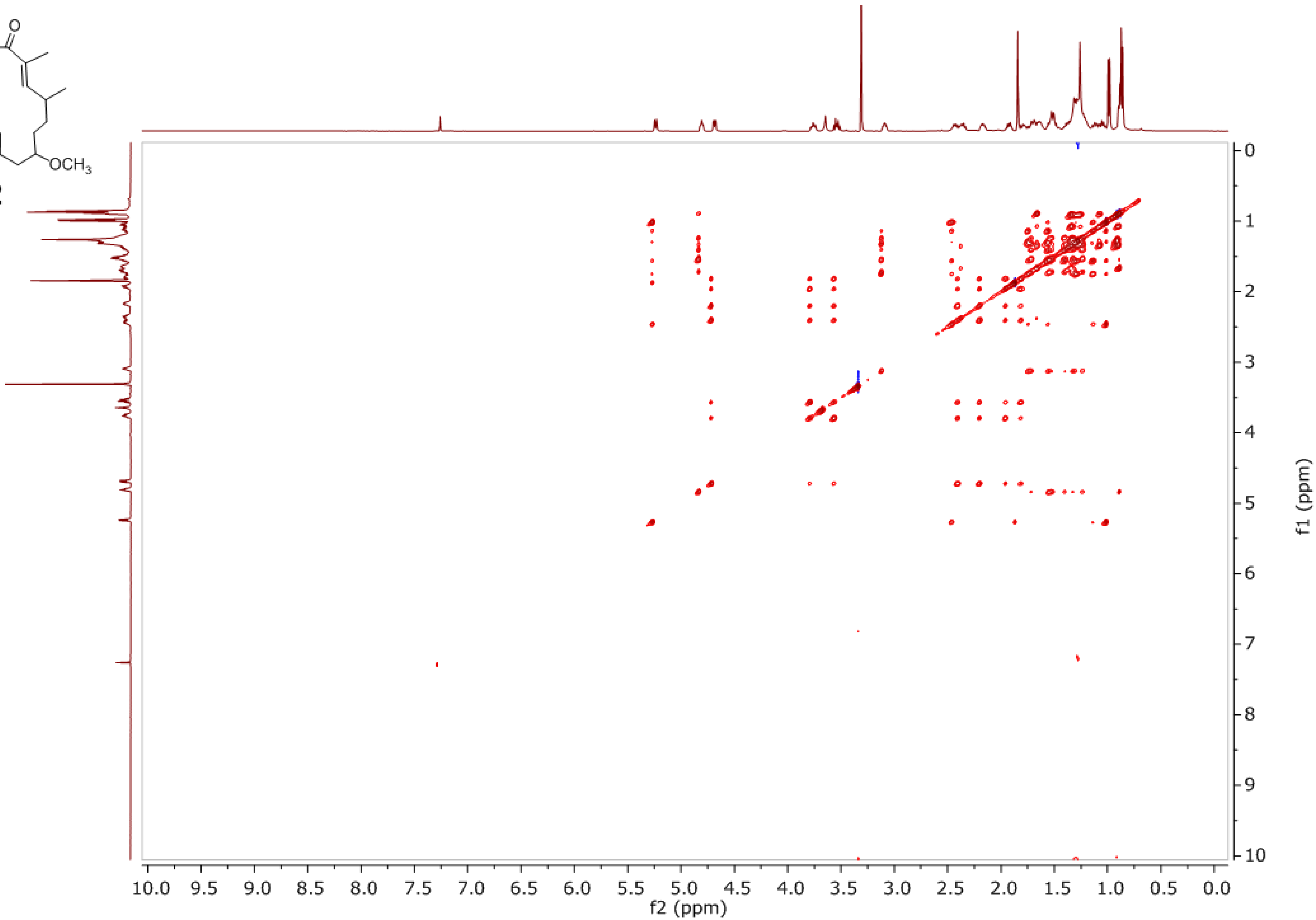

S13. TOCSY spectrum of **2**.

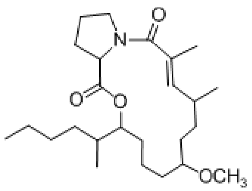

2

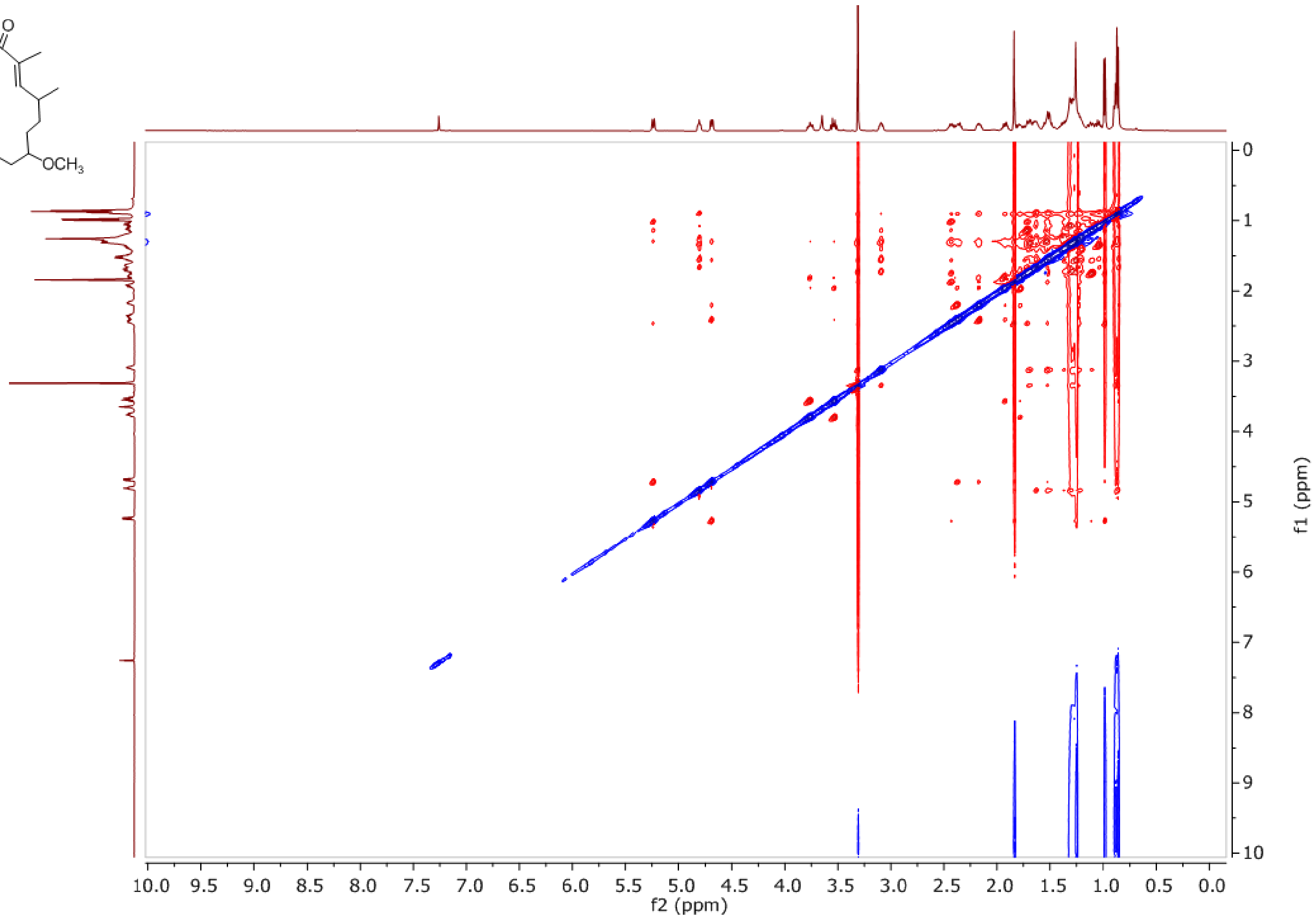

S14. NOESY spectrum of **2**.

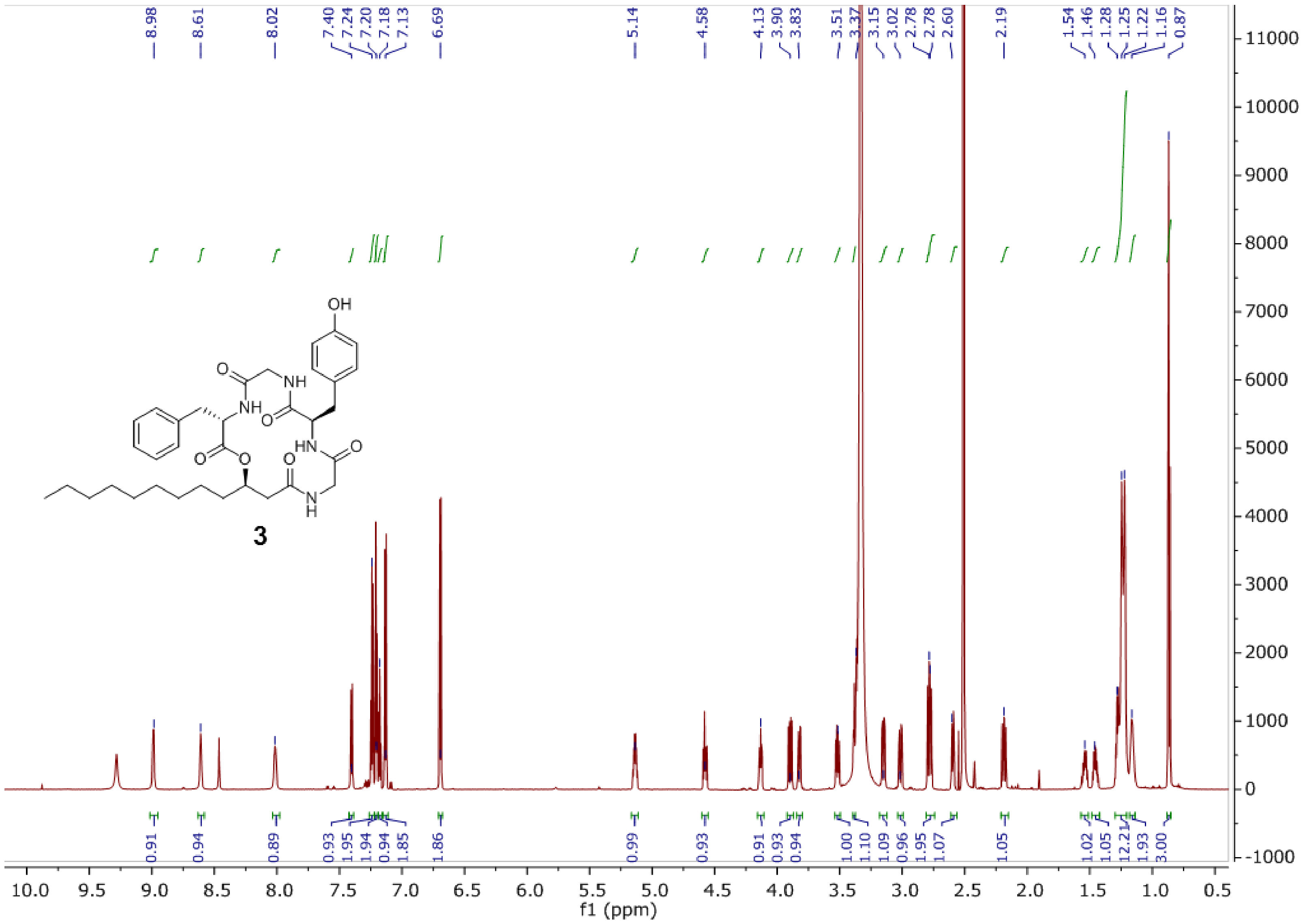

S15. ^{1}H NMR spectrum of unnarmicin D (**3**) (800 MHz, DMSO).

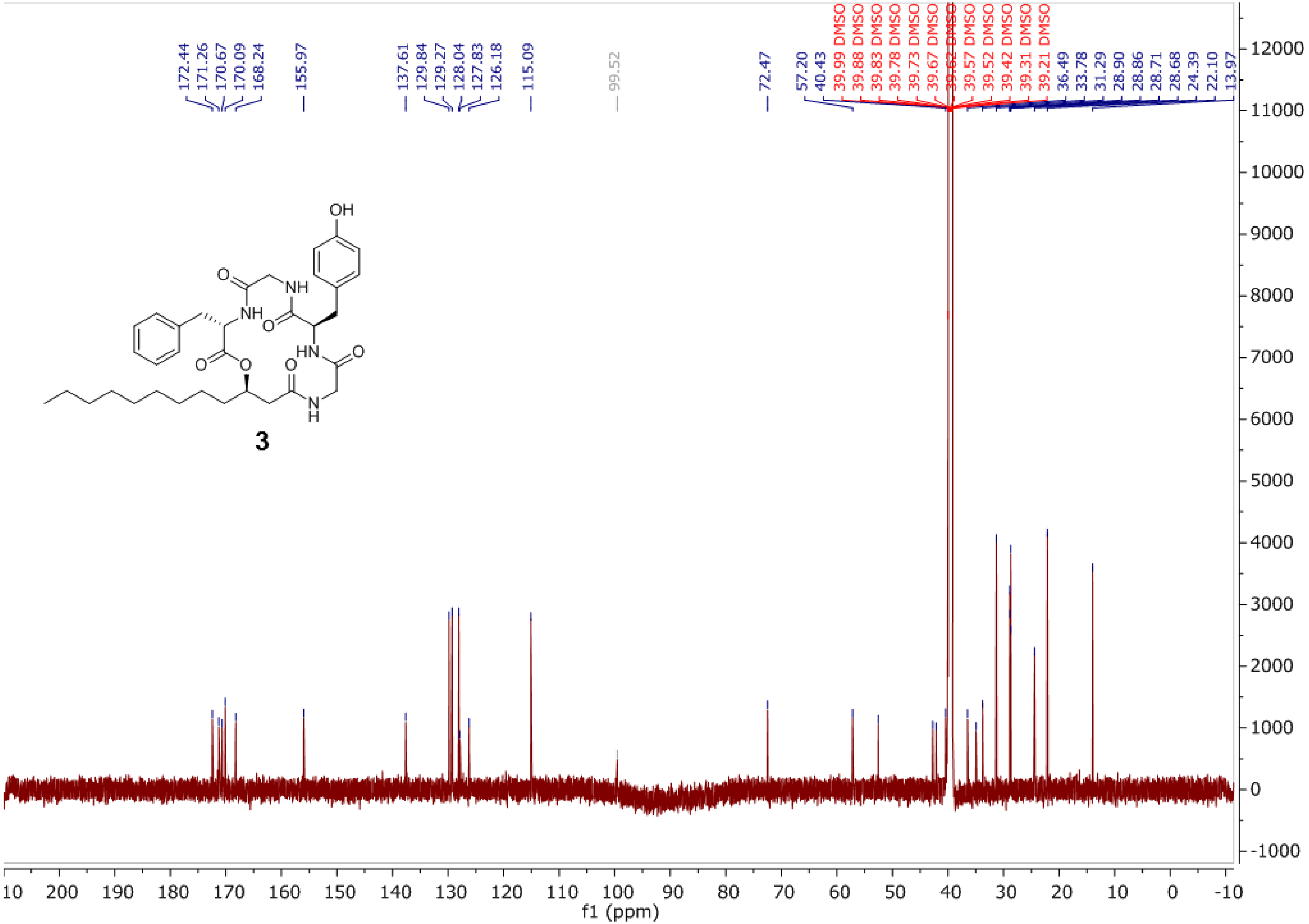

S16. ¹³C NMR spectrum of **3** (200 MHz, DMSO).

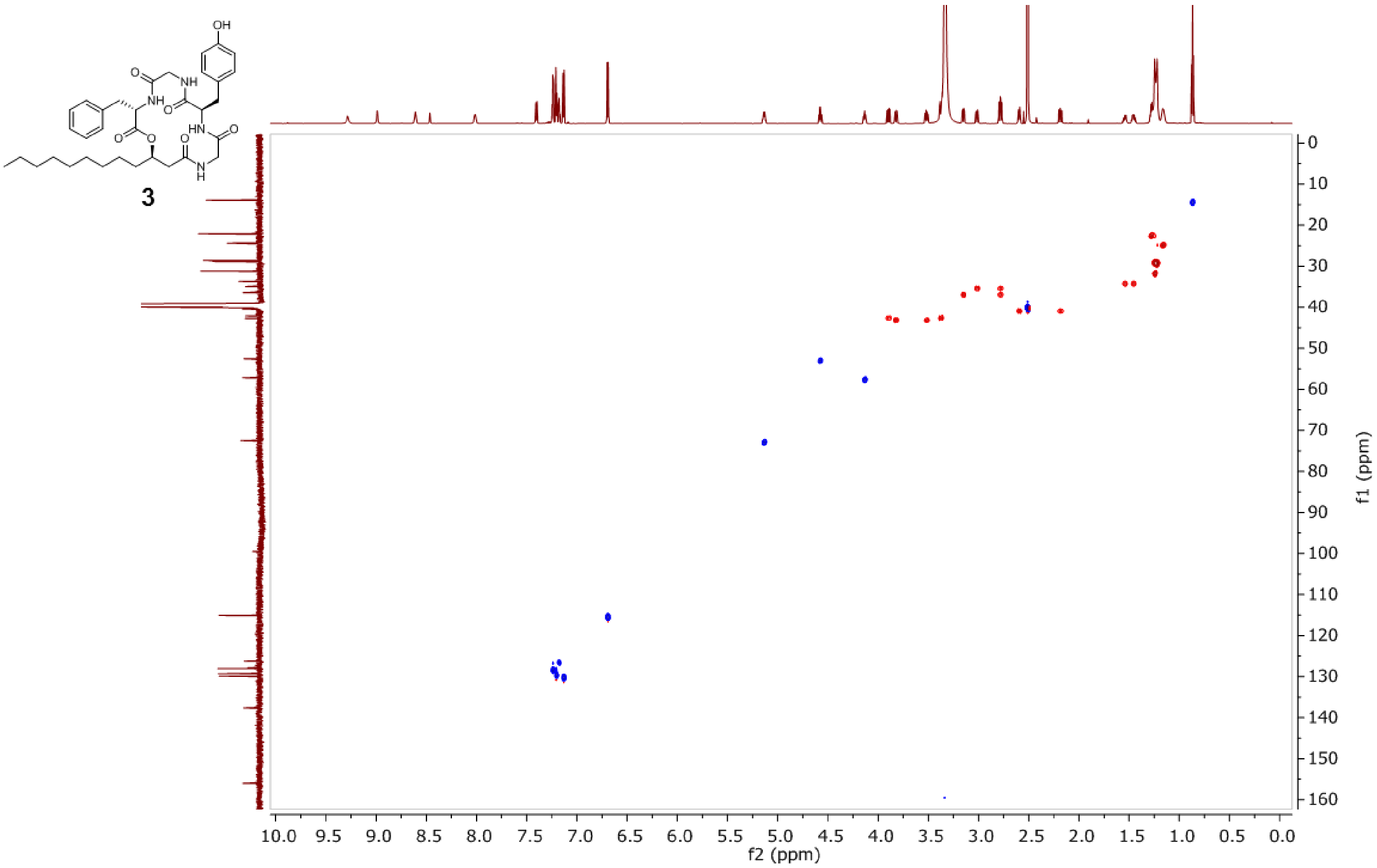

S17. HSQC spectrum of **3**.

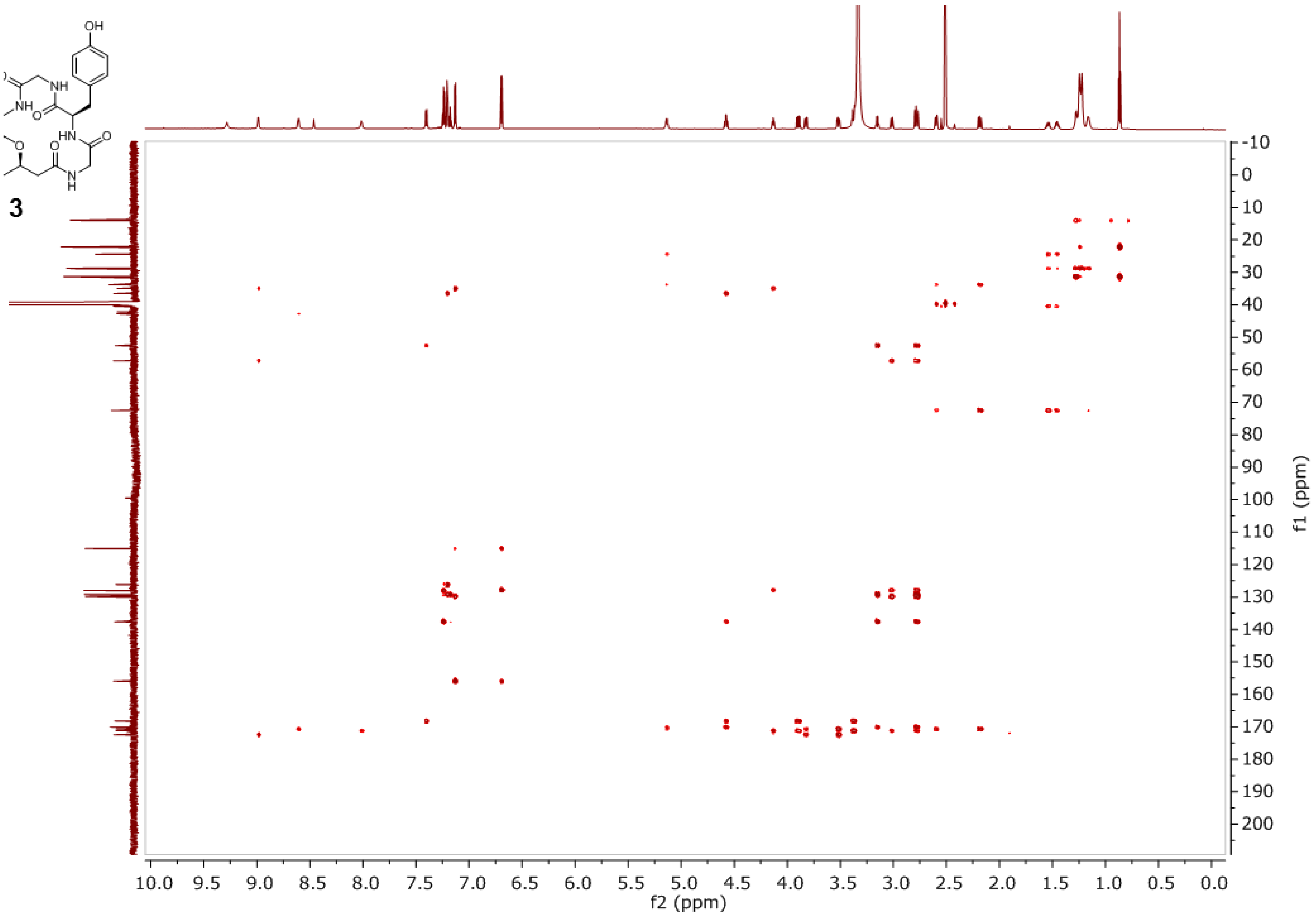

S18. HMBC spectrum of **3**.

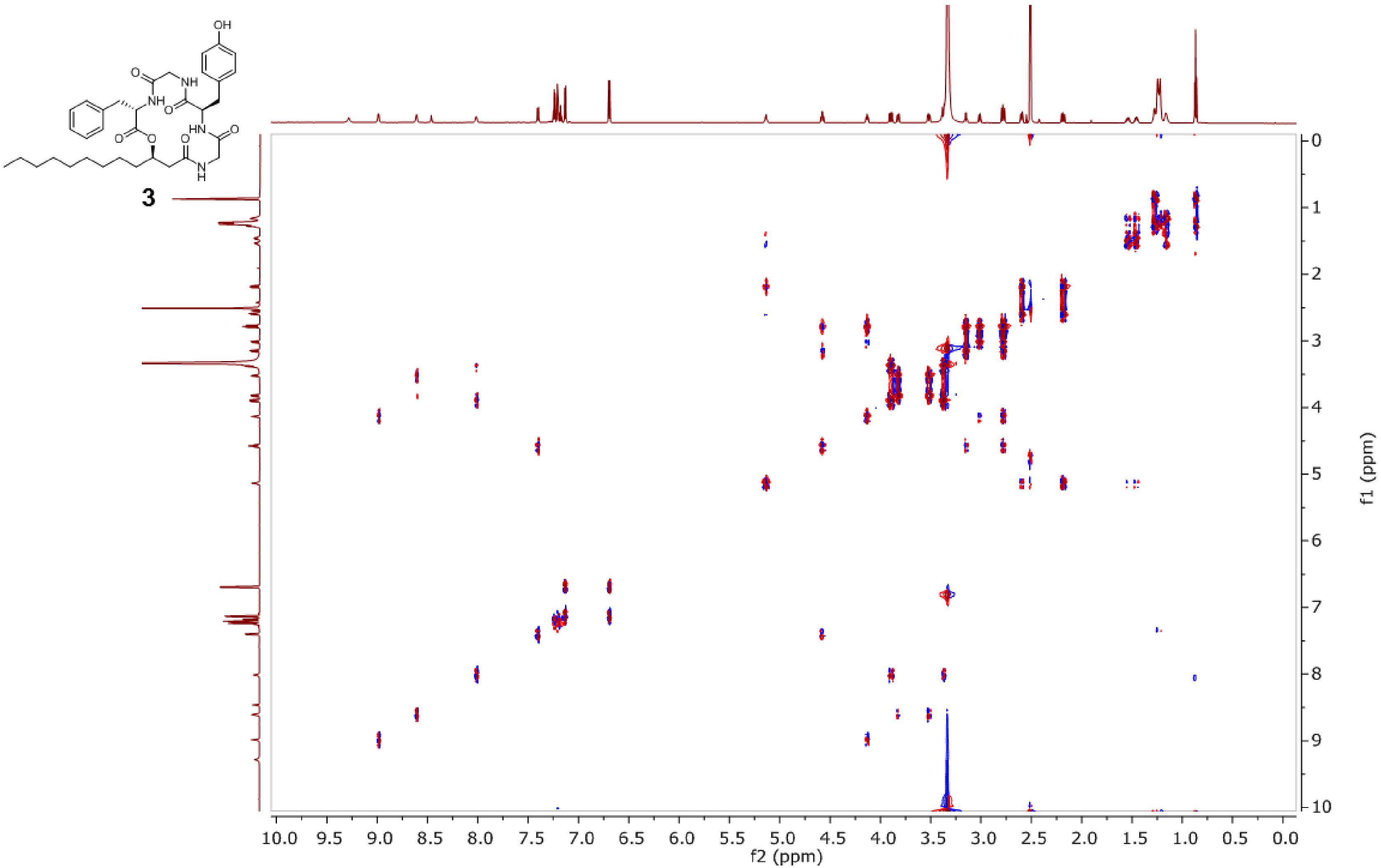

S19. COSY spectrum of **3**.

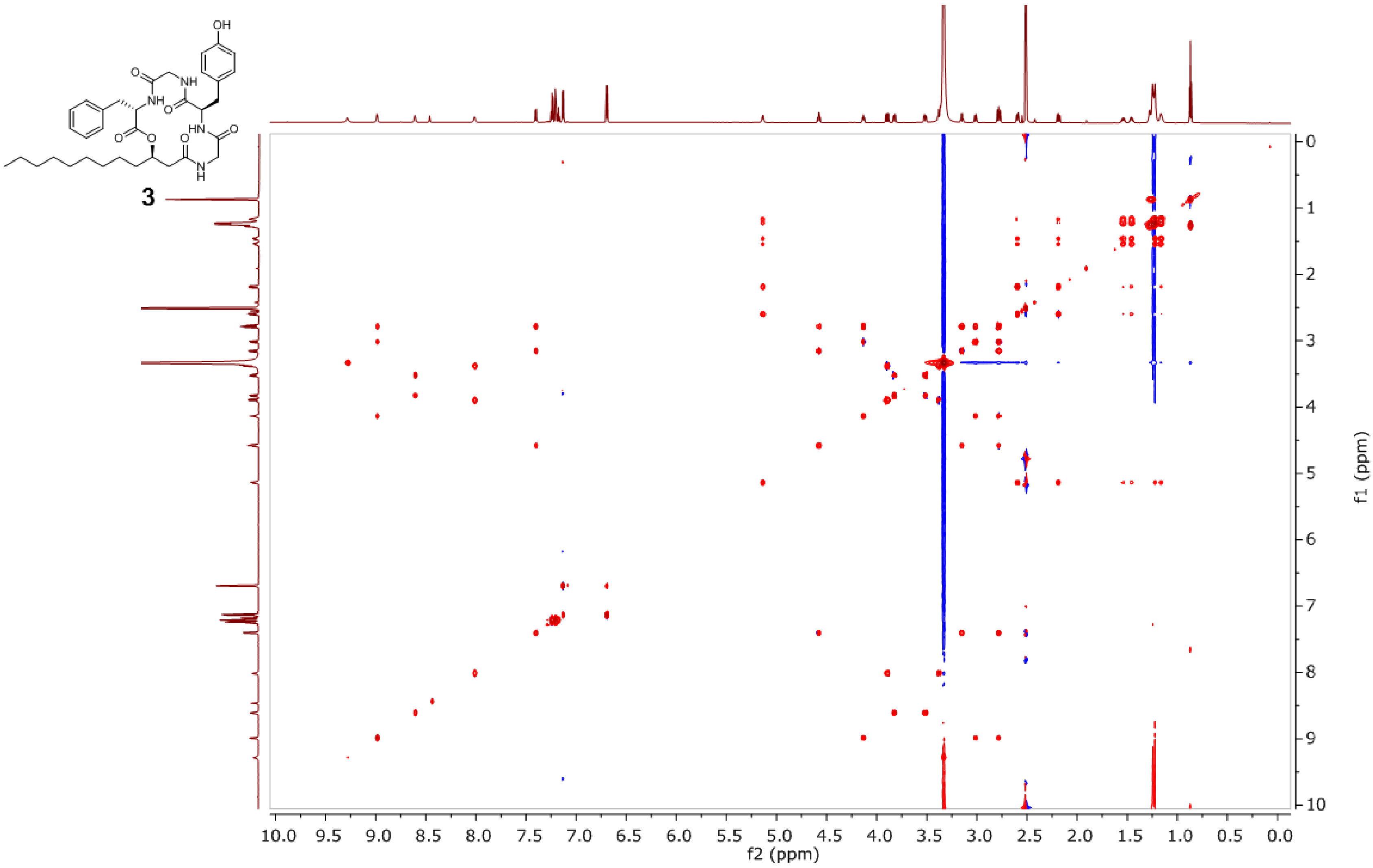

S20. TOCSY spectrum of **3**.

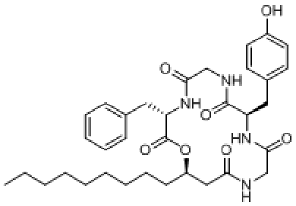

3

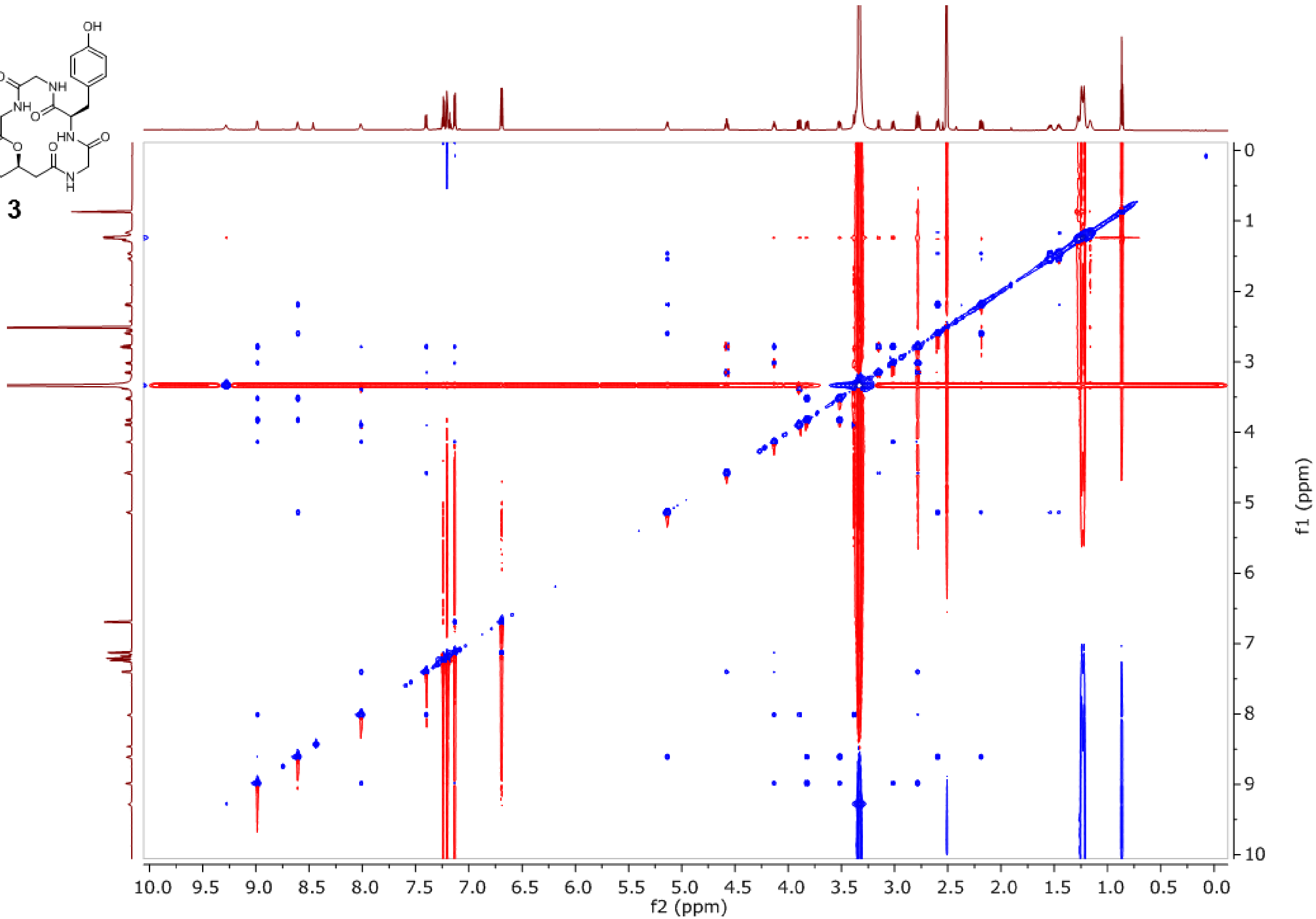

S21. NOESY spectrum of **3**.

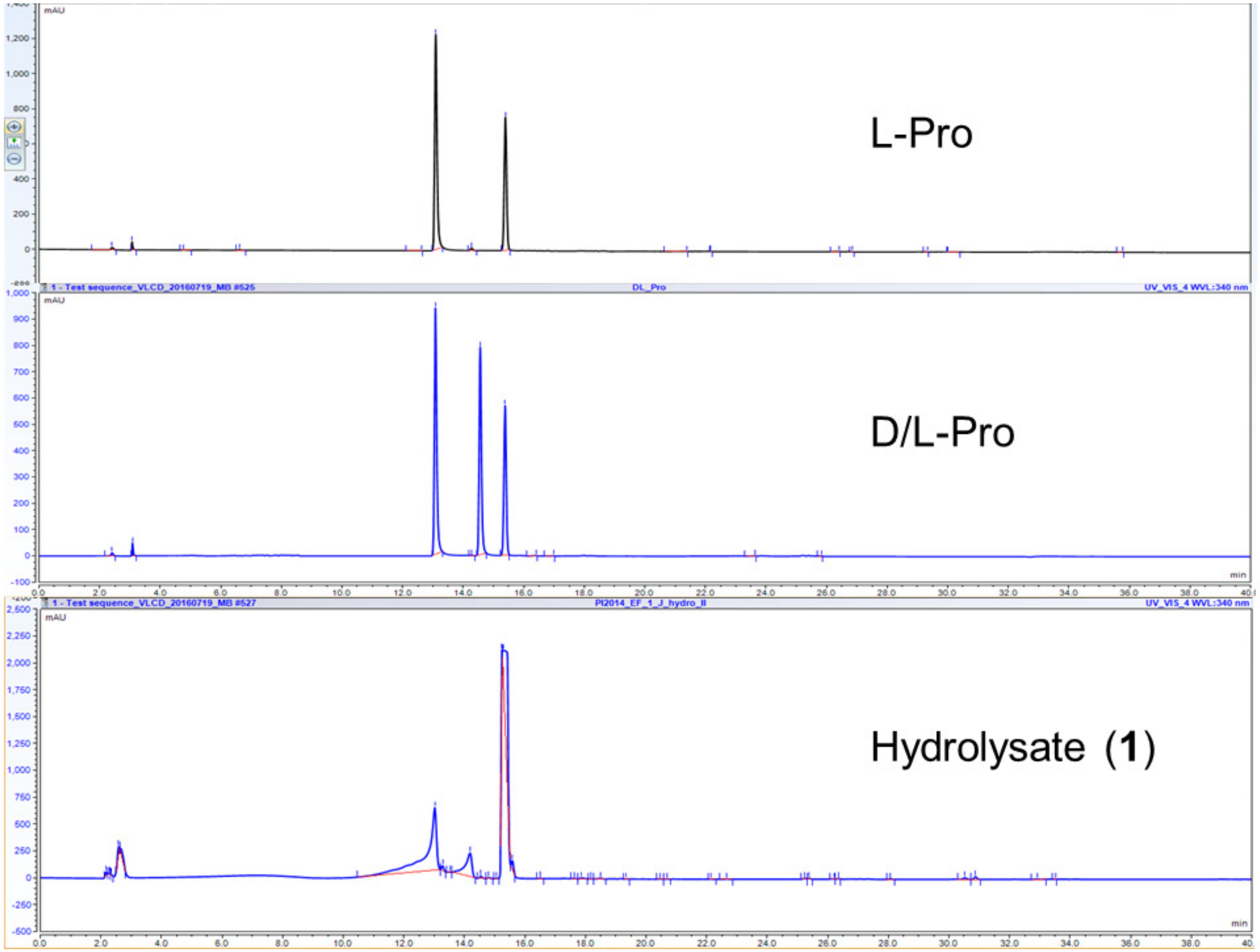

S22. Chromatographic comparison of the L-FDVA reacted acid hydrolyzate of **1** compared to authentic amino acid standards reacted with L-FDVA.

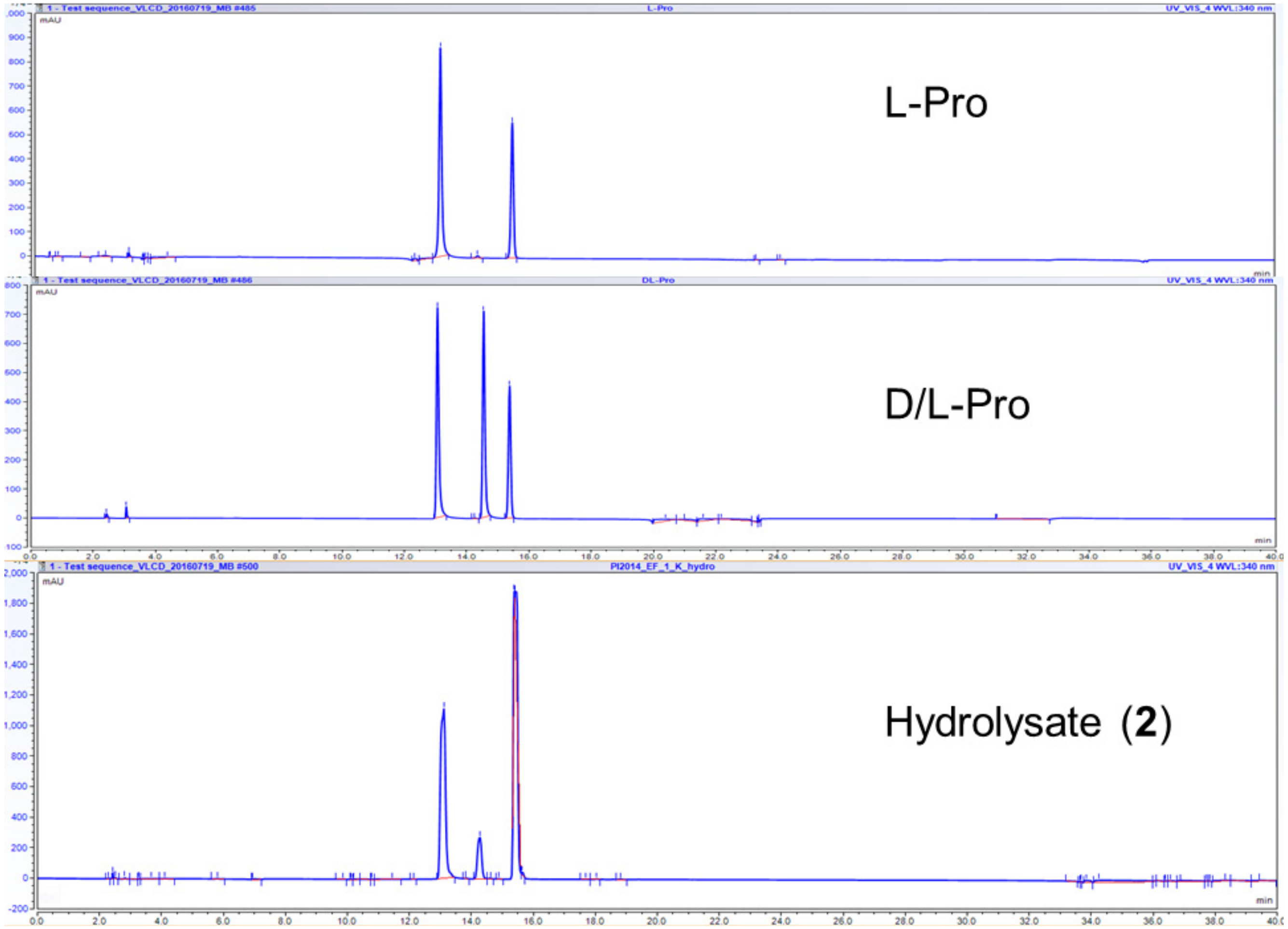

S23. Chromatographic comparison of the L-FDVA reacted acid hydrolyzate of **2** compared to authentic amino acid standards reacted with L-FDVA.

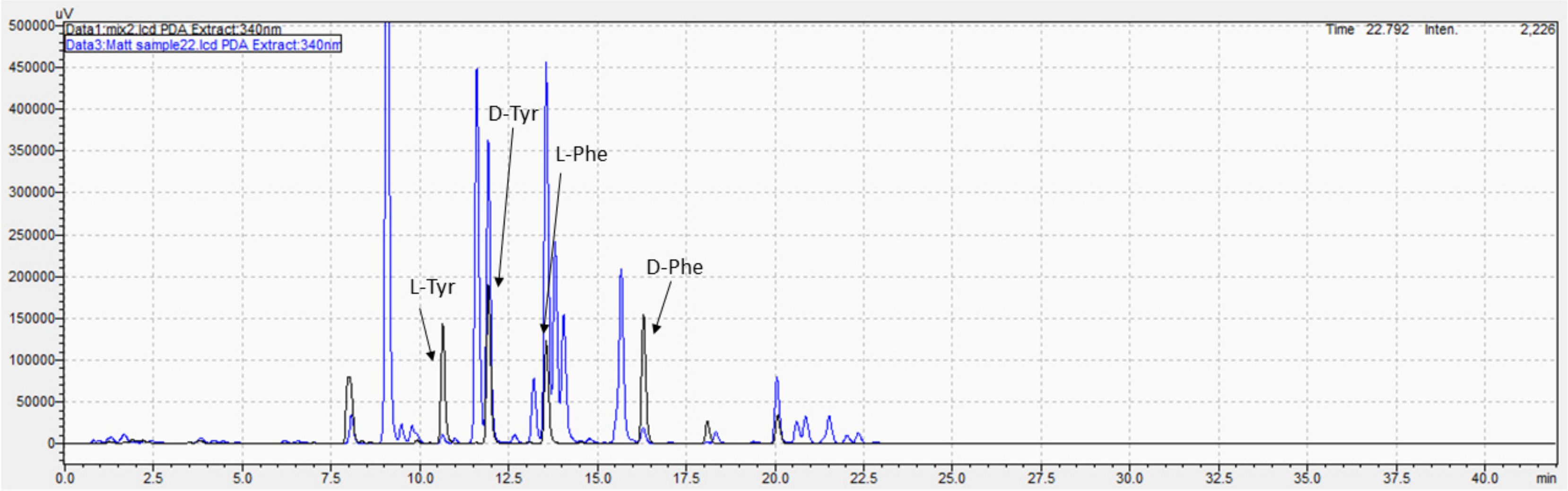

S24. Chromatographic comparison of the L-FDVA reacted acid hydrolyzate of **3** (blue UV trace) compared to authentic amino acid standards reacted with L-FDVA (black UV trace).

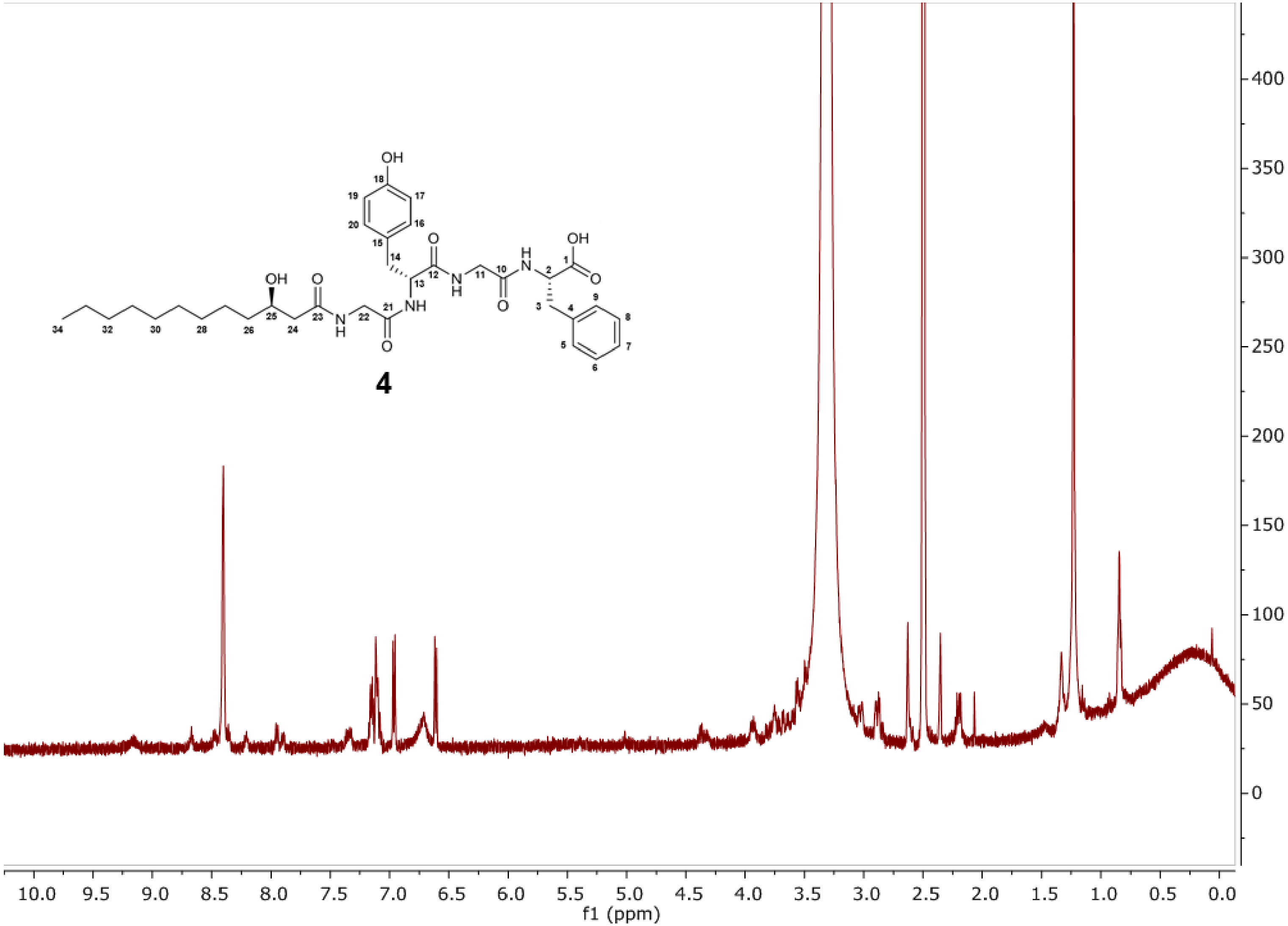

S25. ^{1}H NMR spectrum of unnarmicin D linear derivative (**4**) (500 MHz, DMSO).

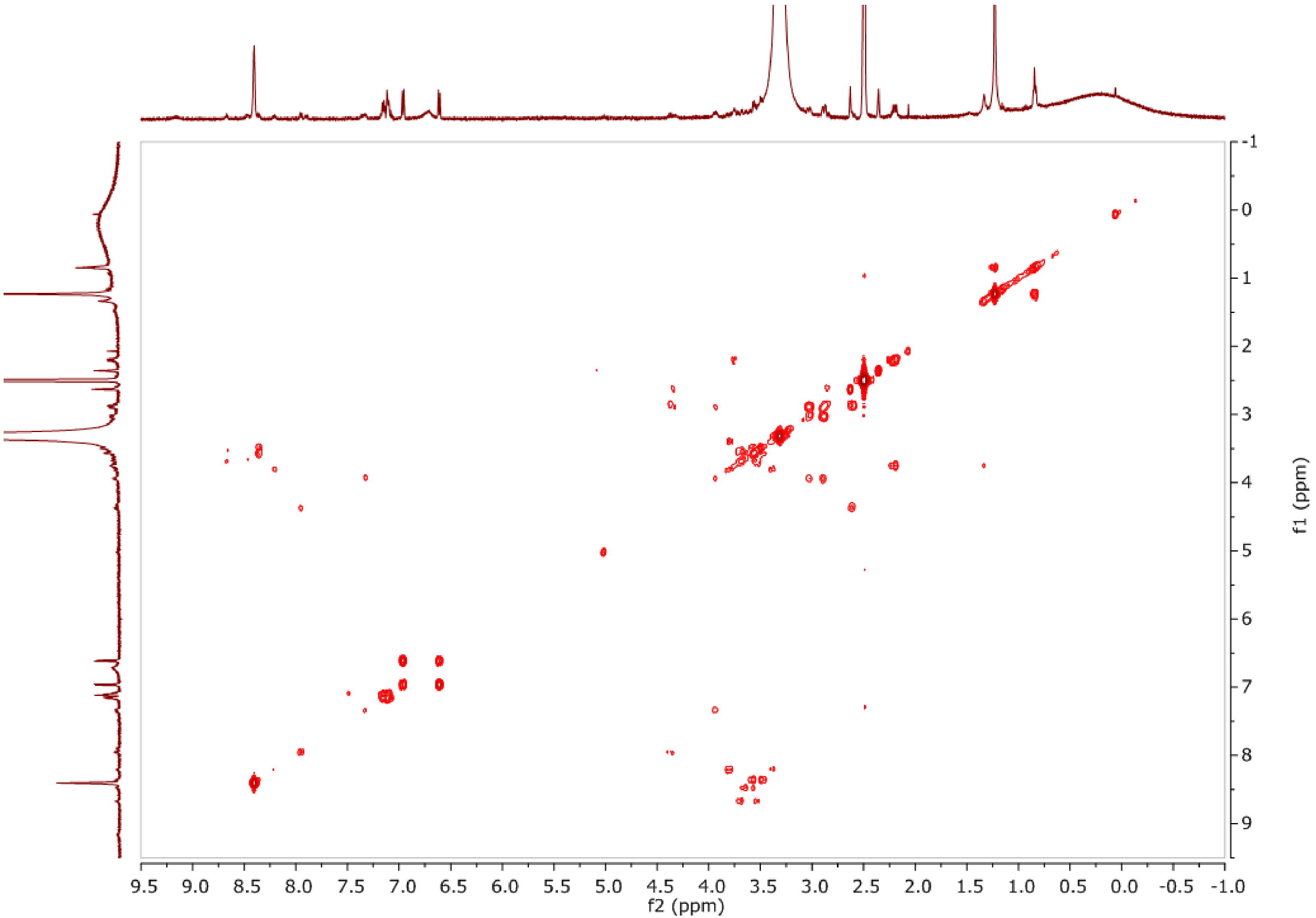

S26. COSY spectrum of unnarmicin D linear derivative (**4**).

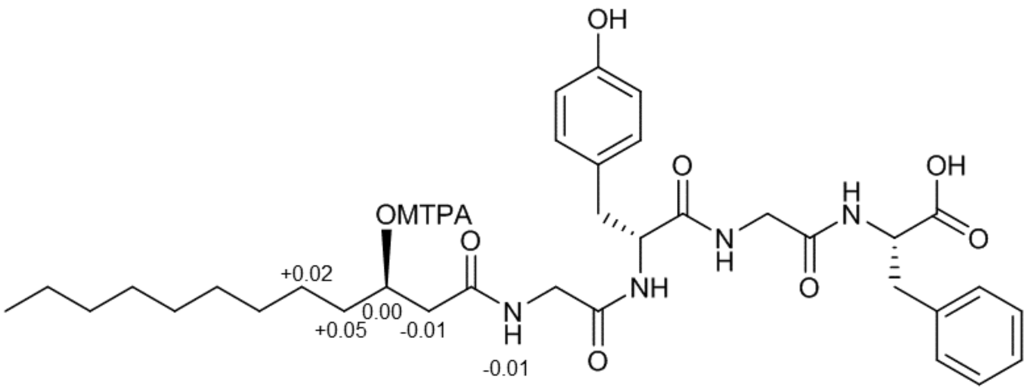

S27. $\Delta(\delta_H S\text{-}\delta_H R)$ values of *S*-MTPA and *R*-MTPA esters of **4**.

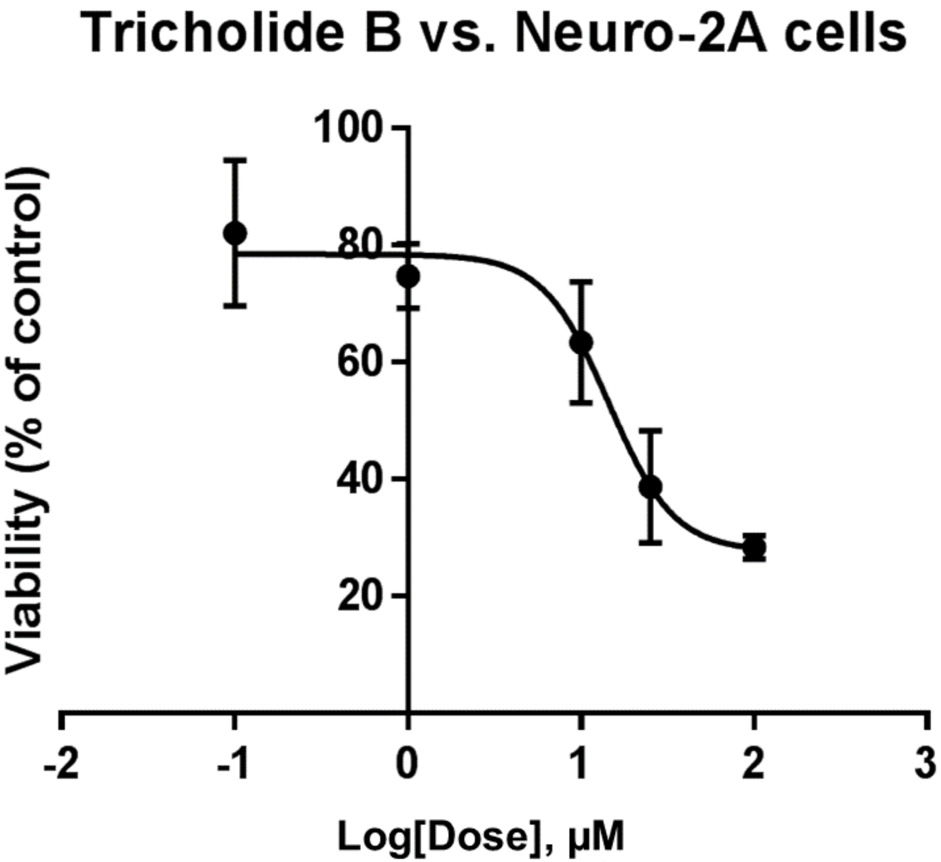

S28. Cytotoxicity of **2** against Neuro-2A mouse neuroblastoma cells. The dosing regime was carried out in triplicate.
